
JOURNAL INFORMATION
==============================
NLM Title Abbreviation: Can Med Educ J
Journal ID: Can Med Educ J
NLM Journal ID: 101560935
Journal ID: CMEJ

Canadian Medical Education Journal

EISSN: 1923-1202
Publisher: University of Saskatchewan

ARTICLE INFORMATION
==============================
PMCID: PMC7162311
DOI: 10.36834/cmej.70106
Article ID: CMEJ-11-e155
Article version: 1
Subjects: Abstracts

Dedicated Poster Abstracts

Electronic publication date: 2020 Apr 18
Publication date: 2020 Apr
Volume: 11
Issue: 2
First page: e155
Last page: e274
Copyright: © 2020; 11(2) licensee Synergies Partners
Copyright year: 2020
License: This is an Open Journal Systems article distributed under the terms of the Creative Commons Attribution License (http://creativecommons.org/licenses/by/2.0) which permits unrestricted use, distribution, and reproduction in any medium, provided the original work is properly cited.
License URL: https://creativecommons.org/licenses/by/2.0/

==============================
JOURNAL INFORMATION
==============================
NLM Title Abbreviation: Can Med Educ J
Journal ID: Can Med Educ J
NLM Journal ID: 101560935
Journal ID: CMEJ

Canadian Medical Education Journal

EISSN: 1923-1202
Publisher: University of Saskatchewan

ARTICLE INFORMATION
==============================
Subjects: Dp 1–1

Medical Student Perceptions of a Case-Based Virtual Dissection Anatomy Curriculum

Bruce B. Forster University of British Columbia

Anique de Bruin Maastricht University

Abigail Arnold University of British Columbia

Rose Hatala University of British Columbia

Jeroen van Merrienboer Maastricht University

Kathryn Darras University of British Columbia

Rebecca Spouge University of British Columbia

Savvas Nicolaou University of British Columbia

Electronic publication date: 2020 Apr 18

JOURNAL INFORMATION
==============================
NLM Title Abbreviation: Can Med Educ J
Journal ID: Can Med Educ J
NLM Journal ID: 101560935
Journal ID: CMEJ

Canadian Medical Education Journal

EISSN: 1923-1202
Publisher: University of Saskatchewan

ARTICLE INFORMATION
==============================
Subjects: Dp 1–2

Using a Digital Multimedia Platform to Teach Anatomical Sciences

Kamal Moghrabi University of British Columbia

Majid Doroudi University of British Columbia

Electronic publication date: 2020 Apr 18

JOURNAL INFORMATION
==============================
NLM Title Abbreviation: Can Med Educ J
Journal ID: Can Med Educ J
NLM Journal ID: 101560935
Journal ID: CMEJ

Canadian Medical Education Journal

EISSN: 1923-1202
Publisher: University of Saskatchewan

ARTICLE INFORMATION
==============================
Subjects: Dp 1–3

Medical students' perceptions of anatomy teaching in the medical undergraduate curriculum: Cadaveric vs radiologic learning

Kathryn Darras University of British Columbia

Juvel Lee University of British Columbia

Anique de Bruin Maastricht University

Savvas Nicolaou University of British Columbia

Bruce Forster University of British Columbia

Electronic publication date: 2020 Apr 18

JOURNAL INFORMATION
==============================
NLM Title Abbreviation: Can Med Educ J
Journal ID: Can Med Educ J
NLM Journal ID: 101560935
Journal ID: CMEJ

Canadian Medical Education Journal

EISSN: 1923-1202
Publisher: University of Saskatchewan

ARTICLE INFORMATION
==============================
Subjects: Dp 1–4

Reimagining dissection lab preparation - the role of digital media in anatomy education

Farnaz Javadian University of British Columbia

Andy Jiang University of British Columbia

Nicole Ng University of British Columbia

Shirley Tse University of British Columbia

Majid Doroudi University of British Columbia

Electronic publication date: 2020 Apr 18

JOURNAL INFORMATION
==============================
NLM Title Abbreviation: Can Med Educ J
Journal ID: Can Med Educ J
NLM Journal ID: 101560935
Journal ID: CMEJ

Canadian Medical Education Journal

EISSN: 1923-1202
Publisher: University of Saskatchewan

ARTICLE INFORMATION
==============================
Subjects: Dp 1–5

Assessing the feasibility of peer-teaching in a first-year medical student virtual dissection curriculum

Nicole Ng University of British Columbia

Andy Jiang University of British Columbia

Bruce Forster University of British Columbia

Savvas Nicolaou University of British Columbia

Jeffrey Hu University of British Columbia

Rebecca Spouge University of British Columbia

James Nugent University of British Columbia

Kathryn Darras University of British Columbia.

Electronic publication date: 2020 Apr 18

JOURNAL INFORMATION
==============================
NLM Title Abbreviation: Can Med Educ J
Journal ID: Can Med Educ J
NLM Journal ID: 101560935
Journal ID: CMEJ

Canadian Medical Education Journal

EISSN: 1923-1202
Publisher: University of Saskatchewan

ARTICLE INFORMATION
==============================
Subjects: Dp 1–6

Integrated virtual and cadaveric dissection laboratories enhance first year medical students' anatomy experience: A pilot study

Kathryn Darras University of British Columbia

Rebecca Spouge University of British Columbia

Rose Hatala University of British Columbia

Savvas Nicolaou University of British Columbia

Jeff Hu University of British Columbia

Anne Worthington University of British Columbia

Claudia Krebs University of British Columbia

Bruce B. Forster University of British Columbia

Electronic publication date: 2020 Apr 18

JOURNAL INFORMATION
==============================
NLM Title Abbreviation: Can Med Educ J
Journal ID: Can Med Educ J
NLM Journal ID: 101560935
Journal ID: CMEJ

Canadian Medical Education Journal

EISSN: 1923-1202
Publisher: University of Saskatchewan

ARTICLE INFORMATION
==============================
Subjects: Dp 1–7

Anatomy in a New Curriculum: Using Digital Media to Facilitate the Learning of Anatomy in the Medical Curriculum

Vishesh Oberoi University of British Columbia

Farshad Hosseini University of British Columbia

Majid Doroudi University of British Columbia

Electronic publication date: 2020 Apr 18

JOURNAL INFORMATION
==============================
NLM Title Abbreviation: Can Med Educ J
Journal ID: Can Med Educ J
NLM Journal ID: 101560935
Journal ID: CMEJ

Canadian Medical Education Journal

EISSN: 1923-1202
Publisher: University of Saskatchewan

ARTICLE INFORMATION
==============================
Subjects: Dp 2–1

OAST at MUN Medicine: An innovative case of service learning and community partnership

Richard Berish Memorial University of Newfoundland

Josh Lehr Memorial University of Newfoundland

Matthew Downer Memorial University of Newfoundland

Luke Duffley Memorial University of Newfoundland

Phil Hillier Memorial University of Newfoundland

Kieran Lacey Memorial University of Newfoundland

Madison Lewis Memorial University of Newfoundland

Brooke Turner Memorial University of Newfoundland

Jill Allison Memorial University of Newfoundland

Electronic publication date: 2020 Apr 18

JOURNAL INFORMATION
==============================
NLM Title Abbreviation: Can Med Educ J
Journal ID: Can Med Educ J
NLM Journal ID: 101560935
Journal ID: CMEJ

Canadian Medical Education Journal

EISSN: 1923-1202
Publisher: University of Saskatchewan

ARTICLE INFORMATION
==============================
Subjects: Dp 2–2

A policy review of social accountability: Implications for evaluation in medical training

Cees van der Vleuten Maastricht University

Jimmie Leppink University of York

Cassandra Barber Maastricht University

Saad Chahine Queen’s University

Electronic publication date: 2020 Apr 18

JOURNAL INFORMATION
==============================
NLM Title Abbreviation: Can Med Educ J
Journal ID: Can Med Educ J
NLM Journal ID: 101560935
Journal ID: CMEJ

Canadian Medical Education Journal

EISSN: 1923-1202
Publisher: University of Saskatchewan

ARTICLE INFORMATION
==============================
Subjects: Dp 2–3

Teaching Medical Learners about the Social Determinants of Health for Improved Patient Treatment at the Bedside

Meghan Gipson University of Toronto

Lisa Richardson MD PhD University of Toronto

Electronic publication date: 2020 Apr 18

JOURNAL INFORMATION
==============================
NLM Title Abbreviation: Can Med Educ J
Journal ID: Can Med Educ J
NLM Journal ID: 101560935
Journal ID: CMEJ

Canadian Medical Education Journal

EISSN: 1923-1202
Publisher: University of Saskatchewan

ARTICLE INFORMATION
==============================
Subjects: Dp 2–4

Voisins de la Rue

Claudianne Laurin Université de Montréal

Marie-Ève Villeneuve Université de Montréal

Camille Dulude Université de Montréal

Electronic publication date: 2020 Apr 18

JOURNAL INFORMATION
==============================
NLM Title Abbreviation: Can Med Educ J
Journal ID: Can Med Educ J
NLM Journal ID: 101560935
Journal ID: CMEJ

Canadian Medical Education Journal

EISSN: 1923-1202
Publisher: University of Saskatchewan

ARTICLE INFORMATION
==============================
Subjects: Dp 2–5

Fostering Advocacy Among Medical Learners Through Community Based Service Learning: A Scoping Study

Jasmine Chahal University of Toronto

Mitesh Patel University of Toronto

Electronic publication date: 2020 Apr 18

JOURNAL INFORMATION
==============================
NLM Title Abbreviation: Can Med Educ J
Journal ID: Can Med Educ J
NLM Journal ID: 101560935
Journal ID: CMEJ

Canadian Medical Education Journal

EISSN: 1923-1202
Publisher: University of Saskatchewan

ARTICLE INFORMATION
==============================
Subjects: Dp 2–6

Evaluating the effectiveness of a 'Muslims in Healthcare' symposium on improving the cultural competence of healthcare trainees

Shaimaa Helal Queen’s University

Emaad Mohammad Queen’s University

Omar Islam Queen’s University

Electronic publication date: 2020 Apr 18

JOURNAL INFORMATION
==============================
NLM Title Abbreviation: Can Med Educ J
Journal ID: Can Med Educ J
NLM Journal ID: 101560935
Journal ID: CMEJ

Canadian Medical Education Journal

EISSN: 1923-1202
Publisher: University of Saskatchewan

ARTICLE INFORMATION
==============================
Subjects: Dp 2–7

A Truth and Reconciliation Commission (TRC) Report Reading Group - Applying TRC's Calls to Action to Medical Students

Aarti Sayal University of Toronto

Electronic publication date: 2020 Apr 18

JOURNAL INFORMATION
==============================
NLM Title Abbreviation: Can Med Educ J
Journal ID: Can Med Educ J
NLM Journal ID: 101560935
Journal ID: CMEJ

Canadian Medical Education Journal

EISSN: 1923-1202
Publisher: University of Saskatchewan

ARTICLE INFORMATION
==============================
Subjects: Dp 2–8

We have work to do! Medical learners and speaking up to positions of power

Jackie Gruber University of Manitoba

Lukas Neville University of Manitoba

Cathy Rocke University of Manitoba

Brenda Elias University of Manitoba

Electronic publication date: 2020 Apr 18

JOURNAL INFORMATION
==============================
NLM Title Abbreviation: Can Med Educ J
Journal ID: Can Med Educ J
NLM Journal ID: 101560935
Journal ID: CMEJ

Canadian Medical Education Journal

EISSN: 1923-1202
Publisher: University of Saskatchewan

ARTICLE INFORMATION
==============================
Subjects: Dp 3–1

Development of the Edmonton Frail Scale Training Course

Hartley Perlmutter University of Alberta

Darryl Rolfson University of Alberta

Thomas Jeffery University of Alberta

Vijay Daniels University of Alberta

Patrick von Hauff University of Alberta

Electronic publication date: 2020 Apr 18

JOURNAL INFORMATION
==============================
NLM Title Abbreviation: Can Med Educ J
Journal ID: Can Med Educ J
NLM Journal ID: 101560935
Journal ID: CMEJ

Canadian Medical Education Journal

EISSN: 1923-1202
Publisher: University of Saskatchewan

ARTICLE INFORMATION
==============================
Subjects: Dp 3–2

Navigating physician education: Curriculum mapping gives new directions to continuing professional development courses

Jessie Thuswaldner University of Ottawa

Heather Lochnan University of Ottawa

Paul Hendry University of Ottawa

Robert Parson University of Ottawa

Electronic publication date: 2020 Apr 18

JOURNAL INFORMATION
==============================
NLM Title Abbreviation: Can Med Educ J
Journal ID: Can Med Educ J
NLM Journal ID: 101560935
Journal ID: CMEJ

Canadian Medical Education Journal

EISSN: 1923-1202
Publisher: University of Saskatchewan

ARTICLE INFORMATION
==============================
Subjects: Dp 3–3

Implementation of a Social Paediatrics Curriculum: Opportunities and Challenges to Cultivating Compassionate Care, Advocacy Skills and Health Equity Understanding

Jacqueline Ogilvie Western University

Breanna Chen Western University

Jill Sangha Children's Hospital, London Health Science Centre

Andrea Ens Western University

Electronic publication date: 2020 Apr 18

JOURNAL INFORMATION
==============================
NLM Title Abbreviation: Can Med Educ J
Journal ID: Can Med Educ J
NLM Journal ID: 101560935
Journal ID: CMEJ

Canadian Medical Education Journal

EISSN: 1923-1202
Publisher: University of Saskatchewan

ARTICLE INFORMATION
==============================
Subjects: Dp 3–4

Curricular Innovations in Postgraduate Psychiatry Training: Lessons Learned from the 'One Room Schoolhouse' Pilot Program at McMaster University

Sarah Payne McMaster University

Sheila Harms McMaster University

Natasha Snelgrove McMaster University

Electronic publication date: 2020 Apr 18

JOURNAL INFORMATION
==============================
NLM Title Abbreviation: Can Med Educ J
Journal ID: Can Med Educ J
NLM Journal ID: 101560935
Journal ID: CMEJ

Canadian Medical Education Journal

EISSN: 1923-1202
Publisher: University of Saskatchewan

ARTICLE INFORMATION
==============================
Subjects: Dp 3–5

Creating an outcome-based curriculum map for a post-graduate medical education residency program

Ryann Kwan Memorial University of Newfoundland

Roger Chafe Memorial University of Newfoundland

Electronic publication date: 2020 Apr 18

JOURNAL INFORMATION
==============================
NLM Title Abbreviation: Can Med Educ J
Journal ID: Can Med Educ J
NLM Journal ID: 101560935
Journal ID: CMEJ

Canadian Medical Education Journal

EISSN: 1923-1202
Publisher: University of Saskatchewan

ARTICLE INFORMATION
==============================
Subjects: Dp 3–6

An evaluation of a pediatrics residency research curriculum

Martha Balicki University of Manitoba

Barr Darja University of Manitoba

Robert Renaud University of Manitoba

Atul Sharma University of Manitoba

Celia Rodd University of Manitoba

Electronic publication date: 2020 Apr 18

JOURNAL INFORMATION
==============================
NLM Title Abbreviation: Can Med Educ J
Journal ID: Can Med Educ J
NLM Journal ID: 101560935
Journal ID: CMEJ

Canadian Medical Education Journal

EISSN: 1923-1202
Publisher: University of Saskatchewan

ARTICLE INFORMATION
==============================
Subjects: Dp 3–7

Development of a Paediatric Residency Professionalism Curriculum

Lauren Friedman University of Toronto

Shannon Willmott University of Toronto

Marie-Pier Lirette University of Toronto

Larissa Shapka University of Toronto

Natalie Jewitt University of Toronto

Gabriel Tse University of Toronto

Amy Zipursky University of Toronto

Lisette Yorke University of Toronto

Adelle Atkinson University of Toronto

Michael Weinstein University of Toronto

Angela Punnett University of Toronto

Electronic publication date: 2020 Apr 18

JOURNAL INFORMATION
==============================
NLM Title Abbreviation: Can Med Educ J
Journal ID: Can Med Educ J
NLM Journal ID: 101560935
Journal ID: CMEJ

Canadian Medical Education Journal

EISSN: 1923-1202
Publisher: University of Saskatchewan

ARTICLE INFORMATION
==============================
Subjects: Dp 3–8

Audit of Radiology Knowledge Among Medical Students in a Problem-Based Learning Curriculum

Senthujan Gunaseelan McMaster University

Danielle Walker McMaster University

Natasha Larocque McMaster University

Electronic publication date: 2020 Apr 18

JOURNAL INFORMATION
==============================
NLM Title Abbreviation: Can Med Educ J
Journal ID: Can Med Educ J
NLM Journal ID: 101560935
Journal ID: CMEJ

Canadian Medical Education Journal

EISSN: 1923-1202
Publisher: University of Saskatchewan

ARTICLE INFORMATION
==============================
Subjects: Dp 3–9

Reviewing non-cancer pain & opioid prescribing curriculum using INSPQ competencies

Michelle Gibson Queen’s University

Portia Tang Queen’s University

Electronic publication date: 2020 Apr 18

JOURNAL INFORMATION
==============================
NLM Title Abbreviation: Can Med Educ J
Journal ID: Can Med Educ J
NLM Journal ID: 101560935
Journal ID: CMEJ

Canadian Medical Education Journal

EISSN: 1923-1202
Publisher: University of Saskatchewan

ARTICLE INFORMATION
==============================
Subjects: Dp 3–1

Improving Medical Education Through Community Service-Learning: Assessing the needs of community organizations involved in service-learning

Ramy Boles University of Toronto

Leshawn Benedict University of Toronto

Joyce Lui McMaster University

Roxanne Wright University of Toronto

Fok Han Leung University of Toronto

Electronic publication date: 2020 Apr 18

JOURNAL INFORMATION
==============================
NLM Title Abbreviation: Can Med Educ J
Journal ID: Can Med Educ J
NLM Journal ID: 101560935
Journal ID: CMEJ

Canadian Medical Education Journal

EISSN: 1923-1202
Publisher: University of Saskatchewan

ARTICLE INFORMATION
==============================
Subjects: Dp 4–1

The novel use of virtual patient cases with expert traces for rare disease education in hematology

Zachary Liederman University of Toronto

Ivan Silver University of Toronto

Rita Selby University of Toronto

Electronic publication date: 2020 Apr 18

JOURNAL INFORMATION
==============================
NLM Title Abbreviation: Can Med Educ J
Journal ID: Can Med Educ J
NLM Journal ID: 101560935
Journal ID: CMEJ

Canadian Medical Education Journal

EISSN: 1923-1202
Publisher: University of Saskatchewan

ARTICLE INFORMATION
==============================
Subjects: Dp 4–2

Clinical teaching unit design: A realist systematic review of evidence-based practices for clinical education and health service delivery

Ryan Sandarage University of British Columbia

Brandon Tang University of British Columbia

Katrina Dutkiewicz University of British Columbia

Stephan Saad University of British Columbia

Jocelyn Chai University of British Columbia

Kristin Dawson University of British Columbia

Vanessa Kitchin University of British Columbia

Rose Hatala University of British Columbia

Iain McCormick University of British Columbia

Barry Kassen University of British Columbia

Electronic publication date: 2020 Apr 18

JOURNAL INFORMATION
==============================
NLM Title Abbreviation: Can Med Educ J
Journal ID: Can Med Educ J
NLM Journal ID: 101560935
Journal ID: CMEJ

Canadian Medical Education Journal

EISSN: 1923-1202
Publisher: University of Saskatchewan

ARTICLE INFORMATION
==============================
Subjects: Dp 4–3

Development of a mobile application to increase motivation, engagement and teaching activity of clinical faculty using gamification principles.

Aazad Abbas University of Toronto

Sarah McClennan University of Toronto

Electronic publication date: 2020 Apr 18

JOURNAL INFORMATION
==============================
NLM Title Abbreviation: Can Med Educ J
Journal ID: Can Med Educ J
NLM Journal ID: 101560935
Journal ID: CMEJ

Canadian Medical Education Journal

EISSN: 1923-1202
Publisher: University of Saskatchewan

ARTICLE INFORMATION
==============================
Subjects: Dp 4–4

Medical Student Comfort Level with the Neurological and Musculoskeletal Examinations

Wasimuddin Syed Queen’s University

Syed Ibrahim Queen’s University

Ali Dergham Queen’s University

Laura Milne Queen’s University

Jessica Trier Queen’s University

Electronic publication date: 2020 Apr 18

JOURNAL INFORMATION
==============================
NLM Title Abbreviation: Can Med Educ J
Journal ID: Can Med Educ J
NLM Journal ID: 101560935
Journal ID: CMEJ

Canadian Medical Education Journal

EISSN: 1923-1202
Publisher: University of Saskatchewan

ARTICLE INFORMATION
==============================
Subjects: Dp 4–5

Pre-Clerkship Procedural Skills Pilot Program

Patrick Albers University of Alberta

Brodie Ritchie University of Alberta

Alexander Miles University of Alberta

Eleanor Crawford University of Alberta

Electronic publication date: 2020 Apr 18

JOURNAL INFORMATION
==============================
NLM Title Abbreviation: Can Med Educ J
Journal ID: Can Med Educ J
NLM Journal ID: 101560935
Journal ID: CMEJ

Canadian Medical Education Journal

EISSN: 1923-1202
Publisher: University of Saskatchewan

ARTICLE INFORMATION
==============================
Subjects: Dp 4–6

A 'systems thinking' conceptual framework to explore clinical practice.

Alan Batt Monash University, Australia

Brett Williams Monash University

Madison Brydges McMaster University

Matthew Leyenaar McMaster University

Walter Tavares University of Toronto

Electronic publication date: 2020 Apr 18

JOURNAL INFORMATION
==============================
NLM Title Abbreviation: Can Med Educ J
Journal ID: Can Med Educ J
NLM Journal ID: 101560935
Journal ID: CMEJ

Canadian Medical Education Journal

EISSN: 1923-1202
Publisher: University of Saskatchewan

ARTICLE INFORMATION
==============================
Subjects: Dp 5–1

Development of a Canadian Program of Continuing Professional Development for Emergency Nurses

Mélanie Marceau Université de Sherbrooke

Denis Bouchard Society of Professionals in Emergency Care (SoPEC)

Monique McLaughlin Society of Professionals in Emergency Care (SoPEC)

Brian Lee Society of Professionals in Emergency Care (SoPEC)

Landon James Society of Professionals in Emergency Care (SoPEC)

Electronic publication date: 2020 Apr 18

JOURNAL INFORMATION
==============================
NLM Title Abbreviation: Can Med Educ J
Journal ID: Can Med Educ J
NLM Journal ID: 101560935
Journal ID: CMEJ

Canadian Medical Education Journal

EISSN: 1923-1202
Publisher: University of Saskatchewan

ARTICLE INFORMATION
==============================
Subjects: Dp 5–2

Evaluation of a Summer Healthcare Improvement Program (SHIP) in Undergraduate Medicine Education

Paul Barber University of Alberta

Jennifer Halasz University of Alberta

Leslie Truong University of Alberta

Kendra Raffael University of Alberta

Christine Phan University of Alberta

Pam Mathura University of Alberta

Narmin Kassam University of Alberta

Tracey Hillier University of Alberta

Jennifer Croden University of Alberta

Electronic publication date: 2020 Apr 18

JOURNAL INFORMATION
==============================
NLM Title Abbreviation: Can Med Educ J
Journal ID: Can Med Educ J
NLM Journal ID: 101560935
Journal ID: CMEJ

Canadian Medical Education Journal

EISSN: 1923-1202
Publisher: University of Saskatchewan

ARTICLE INFORMATION
==============================
Subjects: Dp 5–3

A unified assessment evaluation framework for educational quality improvement

Shujiao Wang McGill

Maryam Wagner McGill

Valérie Dory

Patricia Wade McGill

Beth-Ann Cummings McGill

Electronic publication date: 2020 Apr 18

JOURNAL INFORMATION
==============================
NLM Title Abbreviation: Can Med Educ J
Journal ID: Can Med Educ J
NLM Journal ID: 101560935
Journal ID: CMEJ

Canadian Medical Education Journal

EISSN: 1923-1202
Publisher: University of Saskatchewan

ARTICLE INFORMATION
==============================
Subjects: Dp 5–4

Speaker effectiveness in continuing medical education: a systematic review

Hannah Smith University of Manitoba

Diana Sanchez-Ramirez University of Manitoba

Electronic publication date: 2020 Apr 18

JOURNAL INFORMATION
==============================
NLM Title Abbreviation: Can Med Educ J
Journal ID: Can Med Educ J
NLM Journal ID: 101560935
Journal ID: CMEJ

Canadian Medical Education Journal

EISSN: 1923-1202
Publisher: University of Saskatchewan

ARTICLE INFORMATION
==============================
Subjects: Dp 5–5

Initial evaluation of a new rural mentorship program for preclinical medical students

Jasmine Waslowski University of Toronto

Morag Paton University of Toronto

Mary Freymond University of Toronto

Tristan Brownrigg University of Toronto

Sagar Patel University of Toronto

Shelby Olesovsky University of Toronto

Joyce Nyhof-Young University of Toronto

Electronic publication date: 2020 Apr 18

JOURNAL INFORMATION
==============================
NLM Title Abbreviation: Can Med Educ J
Journal ID: Can Med Educ J
NLM Journal ID: 101560935
Journal ID: CMEJ

Canadian Medical Education Journal

EISSN: 1923-1202
Publisher: University of Saskatchewan

ARTICLE INFORMATION
==============================
Subjects: Dp 5–6

Experiences from a new Women in Medicine club

Adrina Zhong Western University

Elise Quint Western University

Electronic publication date: 2020 Apr 18

JOURNAL INFORMATION
==============================
NLM Title Abbreviation: Can Med Educ J
Journal ID: Can Med Educ J
NLM Journal ID: 101560935
Journal ID: CMEJ

Canadian Medical Education Journal

EISSN: 1923-1202
Publisher: University of Saskatchewan

ARTICLE INFORMATION
==============================
Subjects: Dp 6–1

Sink-or-Swim No More: Development and Implementation of a Shadow Call During Orientation for Incoming Pediatric PGY1 Residents

Tobey Audcent University of Ottawa

Jimin Lee University of Ottawa

Electronic publication date: 2020 Apr 18

JOURNAL INFORMATION
==============================
NLM Title Abbreviation: Can Med Educ J
Journal ID: Can Med Educ J
NLM Journal ID: 101560935
Journal ID: CMEJ

Canadian Medical Education Journal

EISSN: 1923-1202
Publisher: University of Saskatchewan

ARTICLE INFORMATION
==============================
Subjects: Dp 6–2

Food as medicine: How can we teach it?

Andrea Guerin Queen’s University

Victoria Lee-Kim Queen’s University

Karen Grewal Queen’s University

Shannon Willmott University of Toronto

Theresa Nowlan-Suart Queen’s University

Electronic publication date: 2020 Apr 18

JOURNAL INFORMATION
==============================
NLM Title Abbreviation: Can Med Educ J
Journal ID: Can Med Educ J
NLM Journal ID: 101560935
Journal ID: CMEJ

Canadian Medical Education Journal

EISSN: 1923-1202
Publisher: University of Saskatchewan

ARTICLE INFORMATION
==============================
Subjects: Dp 6–3

A situational analysis of pediatric ophthalmology training needs in Ethiopia

Sylvie Bowden University of Toronto

Helen Dimaras The Hospital for Sick Children

Asim Ali The Hospital for Sick Children

Sadik Taju The Hospital for Sick Children

Stephanie Kletke The Hospital for Sick Children

Electronic publication date: 2020 Apr 18

JOURNAL INFORMATION
==============================
NLM Title Abbreviation: Can Med Educ J
Journal ID: Can Med Educ J
NLM Journal ID: 101560935
Journal ID: CMEJ

Canadian Medical Education Journal

EISSN: 1923-1202
Publisher: University of Saskatchewan

ARTICLE INFORMATION
==============================
Subjects: Dp 6–4

Collaborative initiation of the first paediatric ophthalmology fellowship in Eastern Africa

Sylvie Bowden University of Toronto

Helen Dimaras University of Toronto

Max Solish University of Toronto

Electronic publication date: 2020 Apr 18

JOURNAL INFORMATION
==============================
NLM Title Abbreviation: Can Med Educ J
Journal ID: Can Med Educ J
NLM Journal ID: 101560935
Journal ID: CMEJ

Canadian Medical Education Journal

EISSN: 1923-1202
Publisher: University of Saskatchewan

ARTICLE INFORMATION
==============================
Subjects: Dp 6–5

A multimedia approach to enrich learning of developmental milestones in 2nd year medical students

Alanna Roberts Memorial University of Newfoundland

Samantha Stasiuk University of British Columbia

Alasdair Nazerali Maitland University of British Columbia

Electronic publication date: 2020 Apr 18

JOURNAL INFORMATION
==============================
NLM Title Abbreviation: Can Med Educ J
Journal ID: Can Med Educ J
NLM Journal ID: 101560935
Journal ID: CMEJ

Canadian Medical Education Journal

EISSN: 1923-1202
Publisher: University of Saskatchewan

ARTICLE INFORMATION
==============================
Subjects: Dp 6–6

Barriers to Breastfeeding in Physician and Trainee Moms

Alex Frolkis University of Calgary

Allison Michaud University of Calgary

Shannon Ruzycki University of Calgary

Electronic publication date: 2020 Apr 18

JOURNAL INFORMATION
==============================
NLM Title Abbreviation: Can Med Educ J
Journal ID: Can Med Educ J
NLM Journal ID: 101560935
Journal ID: CMEJ

Canadian Medical Education Journal

EISSN: 1923-1202
Publisher: University of Saskatchewan

ARTICLE INFORMATION
==============================
Subjects: Dp 6–7

Impact of stress on the performance of neonatal endotracheal intubation: a randomized controlled trial on manikins.

Ahmed Moussa Université de Montréal

Evelyne Wassef Université de Montréal

Thomas Pennaforte Université de Montréal

Michael-Andrew Assaad Université de Montréal

Electronic publication date: 2020 Apr 18

JOURNAL INFORMATION
==============================
NLM Title Abbreviation: Can Med Educ J
Journal ID: Can Med Educ J
NLM Journal ID: 101560935
Journal ID: CMEJ

Canadian Medical Education Journal

EISSN: 1923-1202
Publisher: University of Saskatchewan

ARTICLE INFORMATION
==============================
Subjects: Dp 6–8

A call to action: Development and Implementation of an Undergraduate Social Paediatric Curriculum

Andrea Ens Western University

Jacqueline Ogilvie Western University

Breanna Chen Western University

Erin Peebles Western University

Tara Mullowney Western University

Electronic publication date: 2020 Apr 18

JOURNAL INFORMATION
==============================
NLM Title Abbreviation: Can Med Educ J
Journal ID: Can Med Educ J
NLM Journal ID: 101560935
Journal ID: CMEJ

Canadian Medical Education Journal

EISSN: 1923-1202
Publisher: University of Saskatchewan

ARTICLE INFORMATION
==============================
Subjects: Dp 7–1

What's the Problem? Collaborating Across Specialties to Develop a Novel PGY-1 Medical Psychiatry Case-Based Curriculum

Natasha Snelgrove McMaster University

Sandra Westcott McMaster University

Sheila Harms McMaster University

Kaif Pardhan McMaster University

Alim Pardhan McMaster University

Michael Brown McMaster University

Electronic publication date: 2020 Apr 18

JOURNAL INFORMATION
==============================
NLM Title Abbreviation: Can Med Educ J
Journal ID: Can Med Educ J
NLM Journal ID: 101560935
Journal ID: CMEJ

Canadian Medical Education Journal

EISSN: 1923-1202
Publisher: University of Saskatchewan

ARTICLE INFORMATION
==============================
Subjects: Dp 7–2

Examining Diagnostic Radiology Residency Case Volumes from a Canadian Perspective: A Marker of Resident Knowledge

Nicholas Cofie Queen’s University

Benjamin Kwan Queen’s University

Benedetto Mussari Queen’s University

Pam Moore Queen’s University

Lynne Meilleur Queen’s University

Omar Islam Queen’s University

Alexandre Menard Queen’s University

Don Soboleski Queen’s University

Nancy Dalgarno Queen’s University

Electronic publication date: 2020 Apr 18

JOURNAL INFORMATION
==============================
NLM Title Abbreviation: Can Med Educ J
Journal ID: Can Med Educ J
NLM Journal ID: 101560935
Journal ID: CMEJ

Canadian Medical Education Journal

EISSN: 1923-1202
Publisher: University of Saskatchewan

ARTICLE INFORMATION
==============================
Subjects: Dp 7–3

Strengths and challenges of the learning environment across postgraduate medical education programs

Loni Desanghere University of Saskatchewan

Anurag Saxena University of Saskatchewan

Scott J. Adams University of Saskatchewan

Electronic publication date: 2020 Apr 18

JOURNAL INFORMATION
==============================
NLM Title Abbreviation: Can Med Educ J
Journal ID: Can Med Educ J
NLM Journal ID: 101560935
Journal ID: CMEJ

Canadian Medical Education Journal

EISSN: 1923-1202
Publisher: University of Saskatchewan

ARTICLE INFORMATION
==============================
Subjects: Dp 7–4

A theoretical model to determine how CaRMS variables impact medical student matching rates.

Andrew Volk University of Alberta

Hollis Lai University of Alberta

Tracey Hillier University of Alberta

Electronic publication date: 2020 Apr 18

JOURNAL INFORMATION
==============================
NLM Title Abbreviation: Can Med Educ J
Journal ID: Can Med Educ J
NLM Journal ID: 101560935
Journal ID: CMEJ

Canadian Medical Education Journal

EISSN: 1923-1202
Publisher: University of Saskatchewan

ARTICLE INFORMATION
==============================
Subjects: Dp 7–5

Assessment of human trafficking knowledge in medical residents prior to and following completion of an online education module

Keon Ma University of Alberta

Jahaan Ali University of Alberta

Julianna Deutscher University of Alberta

Jason Silverman University of Alberta

Chris Novak University of Alberta

Sandy Dong University of Alberta

John Chmelicek University of Alberta

Erica Dance University of Alberta

Helly Goez University of Alberta

Electronic publication date: 2020 Apr 18

JOURNAL INFORMATION
==============================
NLM Title Abbreviation: Can Med Educ J
Journal ID: Can Med Educ J
NLM Journal ID: 101560935
Journal ID: CMEJ

Canadian Medical Education Journal

EISSN: 1923-1202
Publisher: University of Saskatchewan

ARTICLE INFORMATION
==============================
Subjects: Dp 7–6

Witnessed and Experienced forms of Racism in Post-graduate Medical Education at a Canadian Academic Centre

Lara Cooke University of Calgary

Janet de Groot University of Calgary

Lynden Crowshoe University of Calgary

Parichita Choudhury University of Calgary

Electronic publication date: 2020 Apr 18

JOURNAL INFORMATION
==============================
NLM Title Abbreviation: Can Med Educ J
Journal ID: Can Med Educ J
NLM Journal ID: 101560935
Journal ID: CMEJ

Canadian Medical Education Journal

EISSN: 1923-1202
Publisher: University of Saskatchewan

ARTICLE INFORMATION
==============================
Subjects: Dp 7–7

Strategies to Increase Patient Safety Event Reporting By Resident Physicians: A Narrative Review

Ulemu Luhanga Emory University School of Medicine

Maria Aaron Emory University School of Medicine

Adam Webb Emory University School of Medicine

Electronic publication date: 2020 Apr 18

JOURNAL INFORMATION
==============================
NLM Title Abbreviation: Can Med Educ J
Journal ID: Can Med Educ J
NLM Journal ID: 101560935
Journal ID: CMEJ

Canadian Medical Education Journal

EISSN: 1923-1202
Publisher: University of Saskatchewan

ARTICLE INFORMATION
==============================
Subjects: Dp 7–8

Variables qui influencent la rétroaction aux résidents: une recension systématique des écrits

Manon Denis-LeBlanc University of Ottawa

Lyne Pitre University of Ottawa

Maud Mediell University of Ottawa

Adjo David University of Ottawa

Salomon Fotsing University of Ottawa

Eric Dionne University of Ottawa

Electronic publication date: 2020 Apr 18

JOURNAL INFORMATION
==============================
NLM Title Abbreviation: Can Med Educ J
Journal ID: Can Med Educ J
NLM Journal ID: 101560935
Journal ID: CMEJ

Canadian Medical Education Journal

EISSN: 1923-1202
Publisher: University of Saskatchewan

ARTICLE INFORMATION
==============================
Subjects: Dp 8–1

The Impact of Practising Empathy on a Physician's Individual Personhood

Laura Tan Yong Loo Lin School of Medicine, National University of Singapore

Laura Tan Yong Loo Lin School of Medicine, National University of Singapore

Lorraine Tan Yong Loo Lin School of Medicine, National University of Singapore

Yihan Khoo Yong Loo Lin School of Medicine, National University of Singapore

Ying Pin Toh National University Hospital, Singapore

Annelissa Chin Yong Loo Lin School of Medicine, National University of Singapore

Lalit Krishna National Cancer Center Singapore; Yong Loo Lin School of Medicine, National University of Singapore; Center for Biomedical Ethics, National University of Singapore; Duke-NUS Graduate Medical School

Electronic publication date: 2020 Apr 18

JOURNAL INFORMATION
==============================
NLM Title Abbreviation: Can Med Educ J
Journal ID: Can Med Educ J
NLM Journal ID: 101560935
Journal ID: CMEJ

Canadian Medical Education Journal

EISSN: 1923-1202
Publisher: University of Saskatchewan

ARTICLE INFORMATION
==============================
Subjects: Dp 8–2

The Impact of Practising Empathy on a Physician's Relational and Societal Personhood

Laura Tan Yong Loo Lin School of Medicine, National University of Singapore

Lorraine Tan Yong Loo Lin School of Medicine, National University of Singapore

Yihan Khoo Yong Loo Lin School of Medicine, National University of Singapore

Ying Pin Toh National University Hospital, Singapore

Annelissa Chin Yong Loo Lin School of Medicine, National University of Singapore

Lalit Krishna National Cancer Centre Singapore; Yong Loo Lin School of Medicine, National University of Singapore; Centre for Biomedical Ethics, National University of Singapore; Duke-NUS Graduate Medical School

Electronic publication date: 2020 Apr 18

JOURNAL INFORMATION
==============================
NLM Title Abbreviation: Can Med Educ J
Journal ID: Can Med Educ J
NLM Journal ID: 101560935
Journal ID: CMEJ

Canadian Medical Education Journal

EISSN: 1923-1202
Publisher: University of Saskatchewan

ARTICLE INFORMATION
==============================
Subjects: Dp 8–3

Récits de soi récits de soins: Nouvelles perspectives pédagogique et éthique

Annie Descôteaux Université de Montréal

Alexandre Berkesse Centre d'excellence sur le partenariat avec les patients et le public

Marie-Pierre Codsi Université de Montréal

Antoine Payot Université de Montréal

Philippe Karazivan Université de Montréal

Vincent Dumez Université de Montréal

Amélie Du Pont-Thibodeau Université de Montréal

Nathalie Orr-Gaucher Université de Montréal

Melissa Taguemout Université de Montréal

Anne-Sophie Eymard Université de Montréal

Mathieu Jackson Université de Montréal

Clara Dallaire Université de Montréal

Louise Nicaise Université de Montréal

Electronic publication date: 2020 Apr 18

JOURNAL INFORMATION
==============================
NLM Title Abbreviation: Can Med Educ J
Journal ID: Can Med Educ J
NLM Journal ID: 101560935
Journal ID: CMEJ

Canadian Medical Education Journal

EISSN: 1923-1202
Publisher: University of Saskatchewan

ARTICLE INFORMATION
==============================
Subjects: Dp 8–4

“Learning to Play Again”: Medical Student Clowning Elective and Lollipop Moments Day with Molly Penny the Therapeutic Clown at the Children's Hospital of Eastern Ontario (CHEO)

Yipeng Ge University of Ottawa

Ruth Cull Children's Hospital of Eastern Ontario (CHEO)

Electronic publication date: 2020 Apr 18

JOURNAL INFORMATION
==============================
NLM Title Abbreviation: Can Med Educ J
Journal ID: Can Med Educ J
NLM Journal ID: 101560935
Journal ID: CMEJ

Canadian Medical Education Journal

EISSN: 1923-1202
Publisher: University of Saskatchewan

ARTICLE INFORMATION
==============================
Subjects: Dp 8–5

Developing medical students' leadership and collaboration competence through project management in the community

Guylaine Séguin Université de Sherbrooke

Paul Bessette Université de Sherbrooke

Eve-Reine Gagné Université de Sherbrooke

Sylvie Houde Université de Sherbrooke

Sylvie Mathieu Université de Sherbrooke

Sébastien Roulier Université de Sherbrooke

Geneviève Petit Université de Sherbrooke

Linda Pinsonneault Université de Sherbrooke

Ghislaine Houde Université de Sherbrooke

Electronic publication date: 2020 Apr 18

JOURNAL INFORMATION
==============================
NLM Title Abbreviation: Can Med Educ J
Journal ID: Can Med Educ J
NLM Journal ID: 101560935
Journal ID: CMEJ

Canadian Medical Education Journal

EISSN: 1923-1202
Publisher: University of Saskatchewan

ARTICLE INFORMATION
==============================
Subjects: Dp 8–6

Developing the roles of manager and leader for undergraduate medical students in a competency-based education program

Guylaine Séguin Université de Sherbrooke

Paul Bessette Université de Sherbrooke

Gabrielle Trépanier Université de Sherbrooke

Eve-Reine Gagné Université de Sherbrooke

Sylvie Mathieu Université de Sherbrooke

Sylvie Houde Université de Sherbrooke

Damien Bélisle Université de Sherbrooke

Ghislaine Houde Université de Sherbrooke

Electronic publication date: 2020 Apr 18

JOURNAL INFORMATION
==============================
NLM Title Abbreviation: Can Med Educ J
Journal ID: Can Med Educ J
NLM Journal ID: 101560935
Journal ID: CMEJ

Canadian Medical Education Journal

EISSN: 1923-1202
Publisher: University of Saskatchewan

ARTICLE INFORMATION
==============================
Subjects: Dp 8–7

Advocacy in community-based service learning: perspectives of community organizations

Judy Truong University of Toronto

Vanessa Sheng University of Toronto

Priya Sandhu University of Toronto

Yasamin Sadeghi University of Toronto

Electronic publication date: 2020 Apr 18

JOURNAL INFORMATION
==============================
NLM Title Abbreviation: Can Med Educ J
Journal ID: Can Med Educ J
NLM Journal ID: 101560935
Journal ID: CMEJ

Canadian Medical Education Journal

EISSN: 1923-1202
Publisher: University of Saskatchewan

ARTICLE INFORMATION
==============================
Subjects: Dp 9–1

Understanding resident perspectives of critical events during training

Kevin Venus University of Toronto

Nadine Abdullah University of Toronto

Lindsay Melvin University of Toronto

Electronic publication date: 2020 Apr 18

JOURNAL INFORMATION
==============================
NLM Title Abbreviation: Can Med Educ J
Journal ID: Can Med Educ J
NLM Journal ID: 101560935
Journal ID: CMEJ

Canadian Medical Education Journal

EISSN: 1923-1202
Publisher: University of Saskatchewan

ARTICLE INFORMATION
==============================
Subjects: Dp 9–2

Building from the Ground Up: Designing Educational Material Based on Perceived and Unperceived Needs in Malignant Hematology Training in Canadian Hematology Residents

Wilson Lam University of Toronto

Hassan Sibai University of Toronto

Electronic publication date: 2020 Apr 18

JOURNAL INFORMATION
==============================
NLM Title Abbreviation: Can Med Educ J
Journal ID: Can Med Educ J
NLM Journal ID: 101560935
Journal ID: CMEJ

Canadian Medical Education Journal

EISSN: 1923-1202
Publisher: University of Saskatchewan

ARTICLE INFORMATION
==============================
Subjects: Dp 9–3

Resident opinions and educational experience of a Mixed Night-Float System for general surgery resident call

Robin Ralph-Edwards Western University

Michael Ott Western University

Julie Ann VanKoughnett Western University

Electronic publication date: 2020 Apr 18

JOURNAL INFORMATION
==============================
NLM Title Abbreviation: Can Med Educ J
Journal ID: Can Med Educ J
NLM Journal ID: 101560935
Journal ID: CMEJ

Canadian Medical Education Journal

EISSN: 1923-1202
Publisher: University of Saskatchewan

ARTICLE INFORMATION
==============================
Subjects: Dp 9–4

Observation Directe à l'urgence : un milieu favorable?

William Bédard Michel Université de Montréal

Véronique Castonguay Université de Montréal

Pierre Desaulniers Université de Montréal

Éric Piette Université de Montréal

Massimiliano Iseppon Université de Montréal

Electronic publication date: 2020 Apr 18

JOURNAL INFORMATION
==============================
NLM Title Abbreviation: Can Med Educ J
Journal ID: Can Med Educ J
NLM Journal ID: 101560935
Journal ID: CMEJ

Canadian Medical Education Journal

EISSN: 1923-1202
Publisher: University of Saskatchewan

ARTICLE INFORMATION
==============================
Subjects: Dp 9–5

Transitioning towards best practices in residency application and selection, a work in progress: Snapshot of a Canadian university's residency programs admission processes

Patrice Chrétien Raymer Université de Montréal

Jean-Michel Leduc Université de Montréal

Electronic publication date: 2020 Apr 18

JOURNAL INFORMATION
==============================
NLM Title Abbreviation: Can Med Educ J
Journal ID: Can Med Educ J
NLM Journal ID: 101560935
Journal ID: CMEJ

Canadian Medical Education Journal

EISSN: 1923-1202
Publisher: University of Saskatchewan

ARTICLE INFORMATION
==============================
Subjects: Dp 9–6

Pots Maison - une initiative étudiante

Ariane Bergeron Université de Montréal

Judith Lefebvre Université de Montréal

Marie-Ève Villeneuve Université de Montréal

Electronic publication date: 2020 Apr 18

JOURNAL INFORMATION
==============================
NLM Title Abbreviation: Can Med Educ J
Journal ID: Can Med Educ J
NLM Journal ID: 101560935
Journal ID: CMEJ

Canadian Medical Education Journal

EISSN: 1923-1202
Publisher: University of Saskatchewan

ARTICLE INFORMATION
==============================
Subjects: Dp 9–7

Learning to interpret the information gathered through palpation: a meta-analysis

Phillip Surmanowicz University of Alberta

Liam Rourke University of Alberta

Ron Damaant University of Alberta

Electronic publication date: 2020 Apr 18

JOURNAL INFORMATION
==============================
NLM Title Abbreviation: Can Med Educ J
Journal ID: Can Med Educ J
NLM Journal ID: 101560935
Journal ID: CMEJ

Canadian Medical Education Journal

EISSN: 1923-1202
Publisher: University of Saskatchewan

ARTICLE INFORMATION
==============================
Subjects: Dp 9–8

The Role Model Moms Post-Secondary Academy: An MD Program-University-Community Partnership Promoting Higher Education for Mothers Completing Their High School Equivalency

Casey Goldstein University of Limerick

Roxanne Wright University of Toronto

Tianyue Wang McMaster University

Danielle Thibodeau No current affiliation

Joyce Nyhof-Young University of Toronto

Electronic publication date: 2020 Apr 18

JOURNAL INFORMATION
==============================
NLM Title Abbreviation: Can Med Educ J
Journal ID: Can Med Educ J
NLM Journal ID: 101560935
Journal ID: CMEJ

Canadian Medical Education Journal

EISSN: 1923-1202
Publisher: University of Saskatchewan

ARTICLE INFORMATION
==============================
Subjects: Dp 9–9

Key Conceptualizations of Professional Identity Formation: A Scoping Review

Kimberley MacNeil University of British Columbia

Maria Hubinette University of British Columbia

Sandra Jarvis-Selinger University of British Columbia

Electronic publication date: 2020 Apr 18

JOURNAL INFORMATION
==============================
NLM Title Abbreviation: Can Med Educ J
Journal ID: Can Med Educ J
NLM Journal ID: 101560935
Journal ID: CMEJ

Canadian Medical Education Journal

EISSN: 1923-1202
Publisher: University of Saskatchewan

ARTICLE INFORMATION
==============================
Subjects: Dp 10–1

Medical Student Internship Experience: Using Knowledge Translation to Disseminate Lessons Learned

Sara Trincao-Batra University of Ottawa

Kyung Joon Mun University of Toronto

Tracy Pham University of Alberta

Tunde Gondocz Department of Practice Improvement, Canadian Medical Protective Association

Allan McDougall Department of Medical Care Analytics, Canadian Medical Protective Association

Electronic publication date: 2020 Apr 18

JOURNAL INFORMATION
==============================
NLM Title Abbreviation: Can Med Educ J
Journal ID: Can Med Educ J
NLM Journal ID: 101560935
Journal ID: CMEJ

Canadian Medical Education Journal

EISSN: 1923-1202
Publisher: University of Saskatchewan

ARTICLE INFORMATION
==============================
Subjects: Dp 10–2

Decrypting Clerkship: A Novel Resident-Led Program to Facilitate the Transition to Clinical Clerkship

Leah Kosyakovsky University of Toronto

Michael Ruiz University of Toronto

Jonah Himelfarb University of Toronto

Healey Shulman University of Toronto

Mark Bonta University of Toronto

Electronic publication date: 2020 Apr 18

JOURNAL INFORMATION
==============================
NLM Title Abbreviation: Can Med Educ J
Journal ID: Can Med Educ J
NLM Journal ID: 101560935
Journal ID: CMEJ

Canadian Medical Education Journal

EISSN: 1923-1202
Publisher: University of Saskatchewan

ARTICLE INFORMATION
==============================
Subjects: Dp 10–3

Finding Flow: A Systematic Review on Cognitive Flow in Healthcare

Stephanie Jiang Queen’s University

Sydney McQueen University of Toronto

Aidan McParland University of Toronto

Melanie Hammond Mobilio University of Toronto

Carol-Anne Moulton University of Toronto

Electronic publication date: 2020 Apr 18

JOURNAL INFORMATION
==============================
NLM Title Abbreviation: Can Med Educ J
Journal ID: Can Med Educ J
NLM Journal ID: 101560935
Journal ID: CMEJ

Canadian Medical Education Journal

EISSN: 1923-1202
Publisher: University of Saskatchewan

ARTICLE INFORMATION
==============================
Subjects: Dp 10–4

Spiralling Family Medicine Clerkship Curriculum to Enhance Learning using Both Asynchronous and Synchronous Modalities

Keyna Bracken McMaster University

Parth Sharma McMaster University

Emily Allison McMaster University

Electronic publication date: 2020 Apr 18

JOURNAL INFORMATION
==============================
NLM Title Abbreviation: Can Med Educ J
Journal ID: Can Med Educ J
NLM Journal ID: 101560935
Journal ID: CMEJ

Canadian Medical Education Journal

EISSN: 1923-1202
Publisher: University of Saskatchewan

ARTICLE INFORMATION
==============================
Subjects: Dp 10–5

Internal Medicine Enrichment & Development (IMED): early exposure to medicine subspecialties and its influence on students' perceptions of a career as an internist

Kaitlin Endres University of Ottawa

Katina Zheng University of Ottawa

Sarah Elias University of Ottawa

Mimi Deng University of Ottawa

Alexandra Kobza McMaster University

Aimee Li University of Ottawa

Electronic publication date: 2020 Apr 18

JOURNAL INFORMATION
==============================
NLM Title Abbreviation: Can Med Educ J
Journal ID: Can Med Educ J
NLM Journal ID: 101560935
Journal ID: CMEJ

Canadian Medical Education Journal

EISSN: 1923-1202
Publisher: University of Saskatchewan

ARTICLE INFORMATION
==============================
Subjects: Dp 10–6

Redesigned conceptual mapping to support self-regulated learning in problem-based curriculum: from engineering to medical education

Veronique Foley Université de Sherbrooke

David Foley Université de Sherbrooke

Electronic publication date: 2020 Apr 18

JOURNAL INFORMATION
==============================
NLM Title Abbreviation: Can Med Educ J
Journal ID: Can Med Educ J
NLM Journal ID: 101560935
Journal ID: CMEJ

Canadian Medical Education Journal

EISSN: 1923-1202
Publisher: University of Saskatchewan

ARTICLE INFORMATION
==============================
Subjects: Dp 11–1

“Get real”; promoting 'unstandardizable' real patients in OSCEs

Grainne Kearney Queen's University, Belfast

Jenny Johnston Queen's University, Belfast

Nigel Hart Queen's University, Belfast

Gerry Gormley Queen's University, Belfast

Electronic publication date: 2020 Apr 18

JOURNAL INFORMATION
==============================
NLM Title Abbreviation: Can Med Educ J
Journal ID: Can Med Educ J
NLM Journal ID: 101560935
Journal ID: CMEJ

Canadian Medical Education Journal

EISSN: 1923-1202
Publisher: University of Saskatchewan

ARTICLE INFORMATION
==============================
Subjects: Dp 11–2

Minimal Criteria for Lung Ultrasonography

Irene Ma University of Calgary

Janeve Desy University of Calgary

Vicki Noble Cleveland Medical Center

Andrew Liteplo Massachusetts General Hospital

Paul Olszynski University of Saskatchewan

Elaine Dumoulin University of Calgary

Shane Arishenkoff University of British Columbia

Renee Dversdal Oregon Health and Science University

Gigi Liu Johns Hopkins University

Brian Buchanan University of Alberta

Electronic publication date: 2020 Apr 18

JOURNAL INFORMATION
==============================
NLM Title Abbreviation: Can Med Educ J
Journal ID: Can Med Educ J
NLM Journal ID: 101560935
Journal ID: CMEJ

Canadian Medical Education Journal

EISSN: 1923-1202
Publisher: University of Saskatchewan

ARTICLE INFORMATION
==============================
Subjects: Dp 11–3

A Quality Improvement Initiative to Improve Timeliness of Feedback

Sean Borle University of Alberta

Tracey Hillier University of Alberta

Susan Andrew University of Alberta

Cody Surgin University of Alberta

Hollis Lai University of Alberta

Electronic publication date: 2020 Apr 18

JOURNAL INFORMATION
==============================
NLM Title Abbreviation: Can Med Educ J
Journal ID: Can Med Educ J
NLM Journal ID: 101560935
Journal ID: CMEJ

Canadian Medical Education Journal

EISSN: 1923-1202
Publisher: University of Saskatchewan

ARTICLE INFORMATION
==============================
Subjects: Dp 11–4

The Development and Validation of a Simulated Competency Assessment in Diabetic Wound Management

Omar Selim University of Toronto

Andrew Dueck University of Toronto

Catharine Walsh University of Toronto

Ryan Brydges University of Toronto

Mahan Kulasegaram University of Toronto

Allan Okrainec University of Toronto

Electronic publication date: 2020 Apr 18

JOURNAL INFORMATION
==============================
NLM Title Abbreviation: Can Med Educ J
Journal ID: Can Med Educ J
NLM Journal ID: 101560935
Journal ID: CMEJ

Canadian Medical Education Journal

EISSN: 1923-1202
Publisher: University of Saskatchewan

ARTICLE INFORMATION
==============================
Subjects: Dp 11–5

Using Workplace-Based Assessments to Drive Post-Call Feedback: Can It Work?

Amy Lu University of Toronto

Adelle Atkinson University of Toronto

Julie Johnstone University of Toronto

Electronic publication date: 2020 Apr 18

JOURNAL INFORMATION
==============================
NLM Title Abbreviation: Can Med Educ J
Journal ID: Can Med Educ J
NLM Journal ID: 101560935
Journal ID: CMEJ

Canadian Medical Education Journal

EISSN: 1923-1202
Publisher: University of Saskatchewan

ARTICLE INFORMATION
==============================
Subjects: Dp 11–6

Using Kane's Validity Framework to establish evidence for locally Developed MMIs: Implications & Interpretations

Fern Juster McMaster University

Kelly Dore McMaster University

Electronic publication date: 2020 Apr 18

JOURNAL INFORMATION
==============================
NLM Title Abbreviation: Can Med Educ J
Journal ID: Can Med Educ J
NLM Journal ID: 101560935
Journal ID: CMEJ

Canadian Medical Education Journal

EISSN: 1923-1202
Publisher: University of Saskatchewan

ARTICLE INFORMATION
==============================
Subjects: Dp 11–7

Perceptions of Competency; does gender influence self-assessment in UGME?

Helen Mawdsley University of Manitoba

Christen Rachul University of Manitoba

Keevin Bernstein University of Manitoba

Ira Ripstein University of Manitoba

Joanne Hamilton University of Manitoba

Electronic publication date: 2020 Apr 18

JOURNAL INFORMATION
==============================
NLM Title Abbreviation: Can Med Educ J
Journal ID: Can Med Educ J
NLM Journal ID: 101560935
Journal ID: CMEJ

Canadian Medical Education Journal

EISSN: 1923-1202
Publisher: University of Saskatchewan

ARTICLE INFORMATION
==============================
Subjects: Dp 11–8

Un soutien innovateur pour les étudiants qui préparent leur EACMC-1

Véronique Phan Université de Montréal

Stéphane Ouellet Université de Montréal

Dania Ramirez Université de Montréal

Josée Sabourin Université de Montréal

Isabelle Perreault Université de Montréal

Nicolas Fernandez Université de Montréal

Electronic publication date: 2020 Apr 18

JOURNAL INFORMATION
==============================
NLM Title Abbreviation: Can Med Educ J
Journal ID: Can Med Educ J
NLM Journal ID: 101560935
Journal ID: CMEJ

Canadian Medical Education Journal

EISSN: 1923-1202
Publisher: University of Saskatchewan

ARTICLE INFORMATION
==============================
Subjects: Dp 11–9

Performance Report for a 10-Year-Old MD/PhD Program: a Survey of Trainees at the University of Ottawa

Adam Pietrobon University of Ottawa

Lucia Chehadé University of Ottawa

Alexandra Beaudry-Richard University of Ottawa

Brian Keller University of Ottawa

Michael Schlossmacher University of Ottawa

Electronic publication date: 2020 Apr 18

JOURNAL INFORMATION
==============================
NLM Title Abbreviation: Can Med Educ J
Journal ID: Can Med Educ J
NLM Journal ID: 101560935
Journal ID: CMEJ

Canadian Medical Education Journal

EISSN: 1923-1202
Publisher: University of Saskatchewan

ARTICLE INFORMATION
==============================
Subjects: Dp 11–1

Field Notes: Effectiveness and Impact on Family Medicine Residents' Learning in Manitoba

Nicole Zaki University of Manitoba

Teresa Cavett University of Manitoba

Gayle Halas University of Manitoba

Electronic publication date: 2020 Apr 18

JOURNAL INFORMATION
==============================
NLM Title Abbreviation: Can Med Educ J
Journal ID: Can Med Educ J
NLM Journal ID: 101560935
Journal ID: CMEJ

Canadian Medical Education Journal

EISSN: 1923-1202
Publisher: University of Saskatchewan

ARTICLE INFORMATION
==============================
Subjects: Dp 12–1

Discordance Between Competency-Based Assessment Using a Global Versus Reductionist Approach for Medical Students

Holly Caretta-Weyer Stanford University School of Medicine

Electronic publication date: 2020 Apr 18

JOURNAL INFORMATION
==============================
NLM Title Abbreviation: Can Med Educ J
Journal ID: Can Med Educ J
NLM Journal ID: 101560935
Journal ID: CMEJ

Canadian Medical Education Journal

EISSN: 1923-1202
Publisher: University of Saskatchewan

ARTICLE INFORMATION
==============================
Subjects: Dp 12–2

Perceptions of and Barriers to Competency-Based Education

Lindsay Crawford Queen’s University

Sean Taylor Queen’s University

Nicholas Cofie Queen’s University

Damon Dagnone Queen’s University

Laura McEwen Queen’s University

Electronic publication date: 2020 Apr 18

JOURNAL INFORMATION
==============================
NLM Title Abbreviation: Can Med Educ J
Journal ID: Can Med Educ J
NLM Journal ID: 101560935
Journal ID: CMEJ

Canadian Medical Education Journal

EISSN: 1923-1202
Publisher: University of Saskatchewan

ARTICLE INFORMATION
==============================
Subjects: Dp 12–3

Development Of a Competency -Based course for Pre-Cerlkship

Matt Kushneriuk University of Saskatchewan

Deirdre Andres University of Saskatchewan

Andrea Symon University of Saskatchewan

Di Naidu Naidu University of Saskatchewan

Joshua LLoyd University of Saskatchewan

Andrea Symon University of Saskatchewan

Electronic publication date: 2020 Apr 18

JOURNAL INFORMATION
==============================
NLM Title Abbreviation: Can Med Educ J
Journal ID: Can Med Educ J
NLM Journal ID: 101560935
Journal ID: CMEJ

Canadian Medical Education Journal

EISSN: 1923-1202
Publisher: University of Saskatchewan

ARTICLE INFORMATION
==============================
Subjects: Dp 12–4

Perceptions of academic day teaching and barriers to emergency medicine senior residents' attendance

Lisa Thurgur University of Ottawa

Miguel Cortel-LeBlanc University of Ottawa

Jeffrey Landreville University of Ottawa

Electronic publication date: 2020 Apr 18

JOURNAL INFORMATION
==============================
NLM Title Abbreviation: Can Med Educ J
Journal ID: Can Med Educ J
NLM Journal ID: 101560935
Journal ID: CMEJ

Canadian Medical Education Journal

EISSN: 1923-1202
Publisher: University of Saskatchewan

ARTICLE INFORMATION
==============================
Subjects: Dp 12–5

How do healthcare professions develop competency frameworks?

Alan Batt Monash University, Australia

Walter Tavares University of Toronto

Brett Williams Monash University

Electronic publication date: 2020 Apr 18

JOURNAL INFORMATION
==============================
NLM Title Abbreviation: Can Med Educ J
Journal ID: Can Med Educ J
NLM Journal ID: 101560935
Journal ID: CMEJ

Canadian Medical Education Journal

EISSN: 1923-1202
Publisher: University of Saskatchewan

ARTICLE INFORMATION
==============================
Subjects: Dp 12–6

Development of Collaborative Student & UME Course Evaluation

Wenxuan Wang Western University

Michele Weir Western University

Kyle Massey Western University

Gary Tithecott Western University

Electronic publication date: 2020 Apr 18

JOURNAL INFORMATION
==============================
NLM Title Abbreviation: Can Med Educ J
Journal ID: Can Med Educ J
NLM Journal ID: 101560935
Journal ID: CMEJ

Canadian Medical Education Journal

EISSN: 1923-1202
Publisher: University of Saskatchewan

ARTICLE INFORMATION
==============================
Subjects: Dp 12–7

The effect of “active retrieval” on the learning of procedural skills

Cyrus Hamadi Western University

Silvio Ndoja Western University

Charles Dion Western University

Alexander McCarton Western University

Brynn Charron Western University

Marie-Eve LeBel Western University

Electronic publication date: 2020 Apr 18

JOURNAL INFORMATION
==============================
NLM Title Abbreviation: Can Med Educ J
Journal ID: Can Med Educ J
NLM Journal ID: 101560935
Journal ID: CMEJ

Canadian Medical Education Journal

EISSN: 1923-1202
Publisher: University of Saskatchewan

ARTICLE INFORMATION
==============================
Subjects: Dp 12–8

Utility and feasibility of video-based assessment for ureteroscopy and laser lithotripsy

Yuding Wang McMaster University

Kelly dore McMaster University

Edward Matsumoto McMaster University

Dana Russle McMaster University

Nathan Wong MSKCC

Jen Hoogenes McMaster University

Bobby Shayegan McMaster University

Electronic publication date: 2020 Apr 18

JOURNAL INFORMATION
==============================
NLM Title Abbreviation: Can Med Educ J
Journal ID: Can Med Educ J
NLM Journal ID: 101560935
Journal ID: CMEJ

Canadian Medical Education Journal

EISSN: 1923-1202
Publisher: University of Saskatchewan

ARTICLE INFORMATION
==============================
Subjects: Dp 13–1

The Effects of Duty Hours on Wellness Among Resident Physicians in Canada - A Systematic Review

Miranda Wan University of Calgary

Stewart Spence University of Ottawa

Melissa Wan University of British Columbia

Electronic publication date: 2020 Apr 18

JOURNAL INFORMATION
==============================
NLM Title Abbreviation: Can Med Educ J
Journal ID: Can Med Educ J
NLM Journal ID: 101560935
Journal ID: CMEJ

Canadian Medical Education Journal

EISSN: 1923-1202
Publisher: University of Saskatchewan

ARTICLE INFORMATION
==============================
Subjects: Dp 13–2

Reflection on Professional Identity: A Novel Way to Support Resident Wellness

Carly Schenker University of Toronto

Lindsay Herzog University of Toronto

Erin Bearss University of Toronto

Milena Forte University of Toronto

Michael Roberts University of Toronto

Ian Waters University of Toronto

Diana Toubassi University of Toronto

Electronic publication date: 2020 Apr 18

JOURNAL INFORMATION
==============================
NLM Title Abbreviation: Can Med Educ J
Journal ID: Can Med Educ J
NLM Journal ID: 101560935
Journal ID: CMEJ

Canadian Medical Education Journal

EISSN: 1923-1202
Publisher: University of Saskatchewan

ARTICLE INFORMATION
==============================
Subjects: Dp 13–3

Teaching mindfulness-based stress management techniques to medical students: Pilot results from the Simulated Training for Resilience in Various Environments (STRIVE) program

Stephanie Smith University of Calgary

Lauren Griggs University of Calgary

Franco Rizzuti University of Calgary

Joan Horton University of Calgary

Aliya Kassam University of Calgary

Allison Brown University of Calgary

Electronic publication date: 2020 Apr 18

JOURNAL INFORMATION
==============================
NLM Title Abbreviation: Can Med Educ J
Journal ID: Can Med Educ J
NLM Journal ID: 101560935
Journal ID: CMEJ

Canadian Medical Education Journal

EISSN: 1923-1202
Publisher: University of Saskatchewan

ARTICLE INFORMATION
==============================
Subjects: Dp 13–4

Harnessing the power of positive language to promote physician well-being

Marie Leung McMaster University

Olivia Geen McMaster University

Samantha Woodrow Mullett Memorial University of Newfoundland

Meirui Li Western University

Naomi Mudachi University of Toronto

Hely Shah University of Alberta

Electronic publication date: 2020 Apr 18

JOURNAL INFORMATION
==============================
NLM Title Abbreviation: Can Med Educ J
Journal ID: Can Med Educ J
NLM Journal ID: 101560935
Journal ID: CMEJ

Canadian Medical Education Journal

EISSN: 1923-1202
Publisher: University of Saskatchewan

ARTICLE INFORMATION
==============================
Subjects: Dp 13–5

The non-medical use of prescription psychostimulants among medical students of Quebec

Roxanne St-Pierre-Alain Université de Sherbrooke

Maxime Couture Université Laval

Audrey Désilets Université de Montréal

Electronic publication date: 2020 Apr 18

JOURNAL INFORMATION
==============================
NLM Title Abbreviation: Can Med Educ J
Journal ID: Can Med Educ J
NLM Journal ID: 101560935
Journal ID: CMEJ

Canadian Medical Education Journal

EISSN: 1923-1202
Publisher: University of Saskatchewan

ARTICLE INFORMATION
==============================
Subjects: Dp 13–6

Evaluating and reinforcing medical students' engagement with peer-run and faculty-led wellness initiatives

Valerie S. Kim University of Toronto

Allison J. Chen University of Toronto

Anna Chen University of Toronto

Lauren A. Beck University of Toronto

Ivona Berger University of Toronto

Natalie Phung University of Toronto

Caroline Park University of Toronto

Lisa Vi University of Toronto

Electronic publication date: 2020 Apr 18

JOURNAL INFORMATION
==============================
NLM Title Abbreviation: Can Med Educ J
Journal ID: Can Med Educ J
NLM Journal ID: 101560935
Journal ID: CMEJ

Canadian Medical Education Journal

EISSN: 1923-1202
Publisher: University of Saskatchewan

ARTICLE INFORMATION
==============================
Subjects: Dp 13–7

Are the Wellness Benefits of Mindfulness and Resilience Limited by Hidden Motivational Factors in the Learning Environment?

Adam Neufeld University of Saskatchewan

Greg Malin University of Saskatchewan

Electronic publication date: 2020 Apr 18

JOURNAL INFORMATION
==============================
NLM Title Abbreviation: Can Med Educ J
Journal ID: Can Med Educ J
NLM Journal ID: 101560935
Journal ID: CMEJ

Canadian Medical Education Journal

EISSN: 1923-1202
Publisher: University of Saskatchewan

ARTICLE INFORMATION
==============================
Subjects: Dp 13–8

Sentinel Resident Project

Laurence Labine Université de Montréal

Maude Cigna Université de Montréal

Ariane Deneault-Marchand Université de Montréal

Karina Salvo Labelle Université de Montréal

Gabriel Lavoie Université de Montréal

Sarah-Émilie Racine-Hamel Université de Montréal

Sophie Marcoux Université de Montréal

Electronic publication date: 2020 Apr 18

JOURNAL INFORMATION
==============================
NLM Title Abbreviation: Can Med Educ J
Journal ID: Can Med Educ J
NLM Journal ID: 101560935
Journal ID: CMEJ

Canadian Medical Education Journal

EISSN: 1923-1202
Publisher: University of Saskatchewan

ARTICLE INFORMATION
==============================
Subjects: Dp 13–9

Harassment Reporting Mechanisms for Medical Students, Residents and Physicians in Calgary, Alberta: An Environmental Scan

Kendra Martel University of Calgary

Shannon Ruzycki University of Calgary

Electronic publication date: 2020 Apr 18

JOURNAL INFORMATION
==============================
NLM Title Abbreviation: Can Med Educ J
Journal ID: Can Med Educ J
NLM Journal ID: 101560935
Journal ID: CMEJ

Canadian Medical Education Journal

EISSN: 1923-1202
Publisher: University of Saskatchewan

ARTICLE INFORMATION
==============================
Subjects: Dp 14–1

“BEEP-BEEP SIM”: Gamified On-Call Simulation Curriculum for Undergraduate Medical Education

Anthony Seto University of Calgary

William Kennedy University of Calgary

Mackenzie Margetts University of Calgary

Mitchell Rohatensky University of Calgary

Jian Choo University of Calgary

Sean Crooks University of Calgary

Nicole Ertl University of Calgary

Nazia Sharfuddin University of Calgary

Nathan Zondervan University of Calgary

Lucas Streith University of Calgary

Electronic publication date: 2020 Apr 18

JOURNAL INFORMATION
==============================
NLM Title Abbreviation: Can Med Educ J
Journal ID: Can Med Educ J
NLM Journal ID: 101560935
Journal ID: CMEJ

Canadian Medical Education Journal

EISSN: 1923-1202
Publisher: University of Saskatchewan

ARTICLE INFORMATION
==============================
Subjects: Dp 14–2

Application of novel high-fidelity simulation-based tools for the training of digital rectal examination

Louise Hufton Croydon University Hospital

Alejandro Granados

Fernando Bello Imperial College London

Daniel Wheeler Guy's, King's and St Thomas' School of Medical Education, Kings College London, UK

Electronic publication date: 2020 Apr 18

JOURNAL INFORMATION
==============================
NLM Title Abbreviation: Can Med Educ J
Journal ID: Can Med Educ J
NLM Journal ID: 101560935
Journal ID: CMEJ

Canadian Medical Education Journal

EISSN: 1923-1202
Publisher: University of Saskatchewan

ARTICLE INFORMATION
==============================
Subjects: Dp 14–3

Multidisciplinary Healthcare and First Aid Provider Training for In-Flight Medical Emergencies: A Crowdsourcing Session followed by an Airplane Simulation

Anthony Seto University of Calgary

Josh Kariath University of Calgary

Electronic publication date: 2020 Apr 18

JOURNAL INFORMATION
==============================
NLM Title Abbreviation: Can Med Educ J
Journal ID: Can Med Educ J
NLM Journal ID: 101560935
Journal ID: CMEJ

Canadian Medical Education Journal

EISSN: 1923-1202
Publisher: University of Saskatchewan

ARTICLE INFORMATION
==============================
Subjects: Dp 14–4

Novel-Case Learning Environment for Surgical Education

Regan Brownbridge University of Saskatchewan

Amit Persad University of Saskatchewan

Julia Radic University of Saskatchewan

Electronic publication date: 2020 Apr 18

JOURNAL INFORMATION
==============================
NLM Title Abbreviation: Can Med Educ J
Journal ID: Can Med Educ J
NLM Journal ID: 101560935
Journal ID: CMEJ

Canadian Medical Education Journal

EISSN: 1923-1202
Publisher: University of Saskatchewan

ARTICLE INFORMATION
==============================
Subjects: Dp 14–5

Simulating the design and management of a medical clinic

Astrid Bicamumpaka-Shema Université de Montréal

Sarah Zahabi McGill

Sarah Zahabi McGill

Marie-Frédéric Tremblay Université de Montréal

Rosario Rodriguez McGill

Laura Rojas Rozo McGill

Electronic publication date: 2020 Apr 18

JOURNAL INFORMATION
==============================
NLM Title Abbreviation: Can Med Educ J
Journal ID: Can Med Educ J
NLM Journal ID: 101560935
Journal ID: CMEJ

Canadian Medical Education Journal

EISSN: 1923-1202
Publisher: University of Saskatchewan

ARTICLE INFORMATION
==============================
Subjects: Dp 14–6

Speed accuracy tradeoffs in learning to tie surgical knots

Luka Pusic Upper Canada College

Martin Pusic Harvard University

Mark Baxter Upper Canada College

Electronic publication date: 2020 Apr 18

JOURNAL INFORMATION
==============================
NLM Title Abbreviation: Can Med Educ J
Journal ID: Can Med Educ J
NLM Journal ID: 101560935
Journal ID: CMEJ

Canadian Medical Education Journal

EISSN: 1923-1202
Publisher: University of Saskatchewan

ARTICLE INFORMATION
==============================
Subjects: Dp 14–7

Use of a phlebotomy serious game for reducing pre-analytical errors made by blood-drawing health care practitioners

Adam Dubrowski Ontario Tech University (formerly UOIT)

Tim Weber Ontario Tech University (formerly UOIT)

Krystina Clarke Ontario Tech University (formerly UOIT)

Zhujiang Wang Ontario Tech University (formerly UOIT)

Robert Savaglio Ontario Tech University (formerly UOIT)

Ruth Simpson Ontario Tech University (formerly UOIT)

Maurice DiGiuseppe Ontario Tech University (formerly UOIT)

Bill Kapralos Ontario Tech University (formerly UOIT)

Electronic publication date: 2020 Apr 18

JOURNAL INFORMATION
==============================
NLM Title Abbreviation: Can Med Educ J
Journal ID: Can Med Educ J
NLM Journal ID: 101560935
Journal ID: CMEJ

Canadian Medical Education Journal

EISSN: 1923-1202
Publisher: University of Saskatchewan

ARTICLE INFORMATION
==============================
Subjects: Dp 15–1

Sending a Shock through Electroconvulsive Therapy (ECT) Medical Education: Comparing Didactic Seminars-only versus Didactic Seminars Concomitant with High-Fidelity Simulation (HPS) in an Interprofessional Setting Amongst Psychiatry and Anesthesia Trainees

Sarah Frangou University of Saskatchewan

Kristen Edwards University of Saskatchewan

Natalie Garrett University of Saskatchewan

Joshua Frost University of Saskatchewan

Megan Deck University of Saskatchewan

Kiran Rabheru University of Ottawa

Peter Hedlin University of Saskatchewan

Marla Davidson University of Saskatchewan

Electronic publication date: 2020 Apr 18

JOURNAL INFORMATION
==============================
NLM Title Abbreviation: Can Med Educ J
Journal ID: Can Med Educ J
NLM Journal ID: 101560935
Journal ID: CMEJ

Canadian Medical Education Journal

EISSN: 1923-1202
Publisher: University of Saskatchewan

ARTICLE INFORMATION
==============================
Subjects: Dp 15–2

Une activité de simulation de table à des fins de formation interprofessionnelle pour enseigner une nouvelle procédure intra-hospitalière de code rose: une étude pilote exploratoire et rétrospective sur la perception des apprenants

Amélie Frégeau Université de Montréal

Amélie Frégeau Université de Montréal

Alexis Cournoyer Université de Montréal

Nathalie Soucy Université de Montréal

David Fortier Université de Montréal

Pierre Desaulniers Université de Montréal

Véronique Castonguay Université de Montréal

Richard Fleet Université Laval

Electronic publication date: 2020 Apr 18

JOURNAL INFORMATION
==============================
NLM Title Abbreviation: Can Med Educ J
Journal ID: Can Med Educ J
NLM Journal ID: 101560935
Journal ID: CMEJ

Canadian Medical Education Journal

EISSN: 1923-1202
Publisher: University of Saskatchewan

ARTICLE INFORMATION
==============================
Subjects: Dp 15–3

Evaluating the integration of interprofessional collaboration competencies within simulation training

Minisha Suri Canadian Memorial Chiropractic College / St. Michael's Hospital

Deborah Kopansky-Giles University of Toronto

Silvano Mior Canadian Memorial Chiropractic College

Lianne Jeffs University of Toronto

Kari White St. Michael's Hospital

Douglas Campbell St. Michael's Hospital

Electronic publication date: 2020 Apr 18

JOURNAL INFORMATION
==============================
NLM Title Abbreviation: Can Med Educ J
Journal ID: Can Med Educ J
NLM Journal ID: 101560935
Journal ID: CMEJ

Canadian Medical Education Journal

EISSN: 1923-1202
Publisher: University of Saskatchewan

ARTICLE INFORMATION
==============================
Subjects: Dp 15–4

Implementing the 2017 Canadian Opioid Prescribing Guideline within the Thamesview Family Health Team

Jackson Blonde Western University

Andrew Atkins Thamesview Family Health Team

Susan Munro Western University

Electronic publication date: 2020 Apr 18

JOURNAL INFORMATION
==============================
NLM Title Abbreviation: Can Med Educ J
Journal ID: Can Med Educ J
NLM Journal ID: 101560935
Journal ID: CMEJ

Canadian Medical Education Journal

EISSN: 1923-1202
Publisher: University of Saskatchewan

ARTICLE INFORMATION
==============================
Subjects: Dp 15–5

Evaluating the impact of student-led interprofessional musculoskeletal (MSK) workshops on knowledge and confidence of medical students regarding the MSK assessment.

Cyril Boulila McGill

Élise Girouard-Chantal McGill

Matthew Lassman McGill

Christophe Gendron McGill

Timothy Dubé Université de Sherbrooke

Elise Girourard-Chantal McGill

Electronic publication date: 2020 Apr 18

JOURNAL INFORMATION
==============================
NLM Title Abbreviation: Can Med Educ J
Journal ID: Can Med Educ J
NLM Journal ID: 101560935
Journal ID: CMEJ

Canadian Medical Education Journal

EISSN: 1923-1202
Publisher: University of Saskatchewan

ARTICLE INFORMATION
==============================
Subjects: Dp 15–6

The Patient and Family Narratives Seminars at the University of Saskatchewan

Marcel D'Eon University of Saskatchewan

Get Lombamo University of Saskatchewan

Jelyssa Luc University of Saskatchewan

Natasha Hubbard Murdoch Saskatchewan Polytechnic

Poppy Lowe University of Saskatchewan

Electronic publication date: 2020 Apr 18

JOURNAL INFORMATION
==============================
NLM Title Abbreviation: Can Med Educ J
Journal ID: Can Med Educ J
NLM Journal ID: 101560935
Journal ID: CMEJ

Canadian Medical Education Journal

EISSN: 1923-1202
Publisher: University of Saskatchewan

ARTICLE INFORMATION
==============================
Subjects: Dp 15–7

Multi-professional catalyst to the design, research and implementation of state-of-the-art simulation in health professions education

Krystina Clarke Ontario Tech University

Timothy Weber Ontario Tech University

Zhujiang Wang Ontario Tech University

Adam Dubrowski Ontario Tech University

Alvaro Quevedo Ontario Tech University

Bill Kapralos Ontario Tech University

Electronic publication date: 2020 Apr 18

JOURNAL INFORMATION
==============================
NLM Title Abbreviation: Can Med Educ J
Journal ID: Can Med Educ J
NLM Journal ID: 101560935
Journal ID: CMEJ

Canadian Medical Education Journal

EISSN: 1923-1202
Publisher: University of Saskatchewan

ARTICLE INFORMATION
==============================
Subjects: Dp 15–8

Introduction to Biosecurity and Bioterrorism: An Educational Curriculum Response to a Continued Knowledge Threat

Chris Winstead-Derlega Stanford University

Stefanie Sebok-Syer Stanford University

Michelle Bach Stanford University

Elizabeth Trinh Stanford University

Natalia Trounce

Milana Boukhman Stanford University

Electronic publication date: 2020 Apr 18

JOURNAL INFORMATION
==============================
NLM Title Abbreviation: Can Med Educ J
Journal ID: Can Med Educ J
NLM Journal ID: 101560935
Journal ID: CMEJ

Canadian Medical Education Journal

EISSN: 1923-1202
Publisher: University of Saskatchewan

ARTICLE INFORMATION
==============================
Subjects: Dp 15–9

Developing a On-Call Duty Toolkit for Junior Residents at a Cardiology Intensive Care Unit: An Interdisciplinary Collaboration between Nurses and Medical Residents

Camille Pelletier Vernooy Université de Montréal

Clément Cohé Centre hospitalier universitaire de Montréal (CHUM)

Electronic publication date: 2020 Apr 18

JOURNAL INFORMATION
==============================
NLM Title Abbreviation: Can Med Educ J
Journal ID: Can Med Educ J
NLM Journal ID: 101560935
Journal ID: CMEJ

Canadian Medical Education Journal

EISSN: 1923-1202
Publisher: University of Saskatchewan

ARTICLE INFORMATION
==============================
Subjects: Dp 16–1

Queerying the Foundations: an evaluation of the LGBTQ health curriculum in undergraduate medical education at the University of Toronto

Kira Abelsohn University of Toronto

James Owen University of Toronto

Electronic publication date: 2020 Apr 18

JOURNAL INFORMATION
==============================
NLM Title Abbreviation: Can Med Educ J
Journal ID: Can Med Educ J
NLM Journal ID: 101560935
Journal ID: CMEJ

Canadian Medical Education Journal

EISSN: 1923-1202
Publisher: University of Saskatchewan

ARTICLE INFORMATION
==============================
Subjects: Dp 16–2

Decolonizing Evaluation: A Case Study

Cathy Fournier Wilson Centre

David Rojas University of Toronto

Lisa Richardson University of Toronto

Cynthia Whitehead Wilson Centre

Sadie King Women's College Hospital

Electronic publication date: 2020 Apr 18

JOURNAL INFORMATION
==============================
NLM Title Abbreviation: Can Med Educ J
Journal ID: Can Med Educ J
NLM Journal ID: 101560935
Journal ID: CMEJ

Canadian Medical Education Journal

EISSN: 1923-1202
Publisher: University of Saskatchewan

ARTICLE INFORMATION
==============================
Subjects: Dp 16–3

Citizen Forums on Access to Indigenous Health Education: An Innovative Initiative of the Faculté de médecine de l'Université de Montréal

Ahmed Maherzi Université de Montréal

Samuel Blain Université de Montréal

Claudie Paul Regroupement des centres d'amitié autochtones du Québec

Laurianne Petiquay Centre d'amitié autochtone de La Tuque

Claudia Petiquay Centre d'amitié autochtone de Trois-Rivières

Electronic publication date: 2020 Apr 18

JOURNAL INFORMATION
==============================
NLM Title Abbreviation: Can Med Educ J
Journal ID: Can Med Educ J
NLM Journal ID: 101560935
Journal ID: CMEJ

Canadian Medical Education Journal

EISSN: 1923-1202
Publisher: University of Saskatchewan

ARTICLE INFORMATION
==============================
Subjects: Dp 16–4

What makes medical students care? Predictors of interest in Indigenous health learning and clinical practice

Sharon Yeung Queen’s University

Amy Bombay Dalhousie University

Chad Walker Queen’s University

Jeff Denis McMaster University

Debbie Martin Dalhousie University

Paul Sylvestre Queen’s University

Heather Castleden Queen’s University

Electronic publication date: 2020 Apr 18

JOURNAL INFORMATION
==============================
NLM Title Abbreviation: Can Med Educ J
Journal ID: Can Med Educ J
NLM Journal ID: 101560935
Journal ID: CMEJ

Canadian Medical Education Journal

EISSN: 1923-1202
Publisher: University of Saskatchewan

ARTICLE INFORMATION
==============================
Subjects: Dp 16–5

Applying Appreciative Inquiry to Promote Medical Student Diversity

Ashton Cox University of Alberta

Hollis Lai University of Alberta

Tracey Hillier University of Alberta

Joanne Rodger University of Alberta

Thomas Jeffery University of Alberta

Electronic publication date: 2020 Apr 18

JOURNAL INFORMATION
==============================
NLM Title Abbreviation: Can Med Educ J
Journal ID: Can Med Educ J
NLM Journal ID: 101560935
Journal ID: CMEJ

Canadian Medical Education Journal

EISSN: 1923-1202
Publisher: University of Saskatchewan

ARTICLE INFORMATION
==============================
Subjects: Dp 16–6

Transgender Health in Emergency Medicine: A Needs Assessment for Development of Online Curricula

Devon Stride McMaster University

Teresa Chan McMaster University

Electronic publication date: 2020 Apr 18

JOURNAL INFORMATION
==============================
NLM Title Abbreviation: Can Med Educ J
Journal ID: Can Med Educ J
NLM Journal ID: 101560935
Journal ID: CMEJ

Canadian Medical Education Journal

EISSN: 1923-1202
Publisher: University of Saskatchewan

ARTICLE INFORMATION
==============================
Subjects: Dp 16–7

Addressing the need for ethnically diverse stem cell donors in registries globally

Valerie Hladky Université de Montréal

Amelia Lamontagne Université de Montréal

Valerie Hladky Université de Montréal

Amelia Lamontagne Université de Montréal

Qi Li Université de Montréal

Electronic publication date: 2020 Apr 18

JOURNAL INFORMATION
==============================
NLM Title Abbreviation: Can Med Educ J
Journal ID: Can Med Educ J
NLM Journal ID: 101560935
Journal ID: CMEJ

Canadian Medical Education Journal

EISSN: 1923-1202
Publisher: University of Saskatchewan

ARTICLE INFORMATION
==============================
Subjects: Dp 16–8

Get Cultured: A Directory of Inclusive Supports for Black & Indigenous Medical Students at Ontario Medical Schools

Chantal Phillips University of Toronto

Anita Balakrishna University of Toronto

Lisa Robinson University of Toronto

Electronic publication date: 2020 Apr 18

JOURNAL INFORMATION
==============================
NLM Title Abbreviation: Can Med Educ J
Journal ID: Can Med Educ J
NLM Journal ID: 101560935
Journal ID: CMEJ

Canadian Medical Education Journal

EISSN: 1923-1202
Publisher: University of Saskatchewan

ARTICLE INFORMATION
==============================
Subjects: Dp 17–1

Exploring the impact of the Surgical Exploration and Discovery (SEAD) Program on medical students' perceptions of gender biases in surgery: a mixed-method evaluation

Mimi Deng University of Ottawa

Christine Seabrook The Ottawa Hospital

Emily (Tu Nhi) Nham University of Ottawa

James Watterson The Ottawa Hospital

Anahita Malvea University of Ottawa

Electronic publication date: 2020 Apr 18

JOURNAL INFORMATION
==============================
NLM Title Abbreviation: Can Med Educ J
Journal ID: Can Med Educ J
NLM Journal ID: 101560935
Journal ID: CMEJ

Canadian Medical Education Journal

EISSN: 1923-1202
Publisher: University of Saskatchewan

ARTICLE INFORMATION
==============================
Subjects: Dp 17–2

Representation of family medicine in preclerkship clinical cases: Missed opportunities?

Francis Diaz University of Manitoba

Anita Ens University of Manitoba

Sasha Thiem University of Manitoba

Amanda Condon University of Manitoba

Electronic publication date: 2020 Apr 18

JOURNAL INFORMATION
==============================
NLM Title Abbreviation: Can Med Educ J
Journal ID: Can Med Educ J
NLM Journal ID: 101560935
Journal ID: CMEJ

Canadian Medical Education Journal

EISSN: 1923-1202
Publisher: University of Saskatchewan

ARTICLE INFORMATION
==============================
Subjects: Dp 17–3

Assessment of medical student experiences and perceptions of practical surgical teaching throughout UK undergraduate medical education

Daniel Wheeler Guy's, King's and St Thomas' School of Medical Education, King's College London, UK

Filippos Papadopoulos Guy's, King's and St Thomas' School of Medical Education, King's College London, UK

Louise Hufton

Electronic publication date: 2020 Apr 18

JOURNAL INFORMATION
==============================
NLM Title Abbreviation: Can Med Educ J
Journal ID: Can Med Educ J
NLM Journal ID: 101560935
Journal ID: CMEJ

Canadian Medical Education Journal

EISSN: 1923-1202
Publisher: University of Saskatchewan

ARTICLE INFORMATION
==============================
Subjects: Dp 17–4

Addressing Barriers to Learning Geriatric Medicine in Preclerkship

Danielle Portnoy University of Alberta

Martin Moran University of Alberta

Tracey Hillier University of Alberta

Electronic publication date: 2020 Apr 18

JOURNAL INFORMATION
==============================
NLM Title Abbreviation: Can Med Educ J
Journal ID: Can Med Educ J
NLM Journal ID: 101560935
Journal ID: CMEJ

Canadian Medical Education Journal

EISSN: 1923-1202
Publisher: University of Saskatchewan

ARTICLE INFORMATION
==============================
Subjects: Dp 17–5

From Sea to Sea: Undergraduate Medical Education in Medical Genetics

Shaimaa Helal Queen’s University

Andrea Guerin Queen’s University

Hanna Faghfoury Queen’s University

Electronic publication date: 2020 Apr 18

JOURNAL INFORMATION
==============================
NLM Title Abbreviation: Can Med Educ J
Journal ID: Can Med Educ J
NLM Journal ID: 101560935
Journal ID: CMEJ

Canadian Medical Education Journal

EISSN: 1923-1202
Publisher: University of Saskatchewan

ARTICLE INFORMATION
==============================
Subjects: Dp 17–6

The effect of surgical observerships on the perceptions and career choices of preclinical medical students, a mixed methods study

Maureen Thivierge-Southidara Université de Montréal

Mathieu Courchesne Université de Montréal

Steven Bonneau Université de Montréal

Michel Carrier Université de Montréal

Margaret Henri Université de Montréal

Electronic publication date: 2020 Apr 18

JOURNAL INFORMATION
==============================
NLM Title Abbreviation: Can Med Educ J
Journal ID: Can Med Educ J
NLM Journal ID: 101560935
Journal ID: CMEJ

Canadian Medical Education Journal

EISSN: 1923-1202
Publisher: University of Saskatchewan

ARTICLE INFORMATION
==============================
Subjects: Dp 17–7

Review of the Essential Skills Program (ESP) Pilot: A Quality Improvement Project for Developing Surgical Skills

Graham Kasper University of Toronto

Saly Halawa University of Toronto

Shirley Xue Jiang University of Toronto

Caterina Masino University of Toronto

Allan Okrainec University of Toronto

Electronic publication date: 2020 Apr 18

JOURNAL INFORMATION
==============================
NLM Title Abbreviation: Can Med Educ J
Journal ID: Can Med Educ J
NLM Journal ID: 101560935
Journal ID: CMEJ

Canadian Medical Education Journal

EISSN: 1923-1202
Publisher: University of Saskatchewan

ARTICLE INFORMATION
==============================
Subjects: Dp 17–8

A logic model for IMed: An Internal Medicine Summer Exploration Program for Pre-Clerkship Students

Alexandra Kobza University of Ottawa

Kaitlin Endres University of Ottawa

Katina Zheng University of Ottawa

Sarah Elias University of Ottawa

Mimi Deng University of Ottawa

Aimee Li University of Ottawa

Electronic publication date: 2020 Apr 18

JOURNAL INFORMATION
==============================
NLM Title Abbreviation: Can Med Educ J
Journal ID: Can Med Educ J
NLM Journal ID: 101560935
Journal ID: CMEJ

Canadian Medical Education Journal

EISSN: 1923-1202
Publisher: University of Saskatchewan

ARTICLE INFORMATION
==============================
Subjects: Dp 18–1

The use of video-based material as preparation for Problem-Assisted Learning (PAL) during the Internal Medicine Clerkship Rotation at the University of Ottawa Medical School

Michelle Turcotte University of Ottawa

Chloé Thabet University of Ottawa

Melissa Phuong University of Ottawa

Glara Rhee McMaster University

Phillip Tsang University of Ottawa

Olwyn Foley Université de Sherbrooke

Ryan Gotfrit University of Ottawa

Laura Zuccaro University of Toronto

Vladimir Contreras-Dominguez University of Ottawa

Robert Bell University of Ottawa

Electronic publication date: 2020 Apr 18

JOURNAL INFORMATION
==============================
NLM Title Abbreviation: Can Med Educ J
Journal ID: Can Med Educ J
NLM Journal ID: 101560935
Journal ID: CMEJ

Canadian Medical Education Journal

EISSN: 1923-1202
Publisher: University of Saskatchewan

ARTICLE INFORMATION
==============================
Subjects: Dp 18–2

To Lecture or Not to Lecture, That is The Question! Modern Medical and Nursing Student Perceptions Regarding Lectures and Lecture Attendance at the University of Ottawa

Safaa El Bialy University of Ottawa

Mohammad Jay University of Ottawa

John Leddy University of Ottawa

Christopher Ramnanan University of Ottawa

Yamilee Hebert University of Ottawa

Dalia limor Karol University of Ottawa

Neraj Manhas University of Ottawa

Electronic publication date: 2020 Apr 18

JOURNAL INFORMATION
==============================
NLM Title Abbreviation: Can Med Educ J
Journal ID: Can Med Educ J
NLM Journal ID: 101560935
Journal ID: CMEJ

Canadian Medical Education Journal

EISSN: 1923-1202
Publisher: University of Saskatchewan

ARTICLE INFORMATION
==============================
Subjects: Dp 18–3

Fostering Intergenerational Education: An Experiential Learning Program for Medical Students and Older Adults

Rebecca Correia McMaster University

Lindsay Klea McMaster University

Graham Campbell McMaster University

Andrew Costa McMaster University

Electronic publication date: 2020 Apr 18

JOURNAL INFORMATION
==============================
NLM Title Abbreviation: Can Med Educ J
Journal ID: Can Med Educ J
NLM Journal ID: 101560935
Journal ID: CMEJ

Canadian Medical Education Journal

EISSN: 1923-1202
Publisher: University of Saskatchewan

ARTICLE INFORMATION
==============================
Subjects: Dp 18–4

Assessing performance of Endocrinology & Metabolism Residents after wearing diabetes technology: A matter of experience

Linda Wang University of Ottawa

Stephanie Dizon University of Ottawa

Amel Arnaout University of Ottawa

Electronic publication date: 2020 Apr 18

JOURNAL INFORMATION
==============================
NLM Title Abbreviation: Can Med Educ J
Journal ID: Can Med Educ J
NLM Journal ID: 101560935
Journal ID: CMEJ

Canadian Medical Education Journal

EISSN: 1923-1202
Publisher: University of Saskatchewan

ARTICLE INFORMATION
==============================
Subjects: Dp 18–5

Mixed methods analysis of an automated e-mail audit and feedback intervention for fostering (emergency) physician reflection

William Kennedy University of Calgary

Daniel Andruchow University of Calgary

Shawn Dowling University of Calgary

Kevin Lonergan Alberta Health Services

Tom Rich Alberta Health Services

Catherine Patocka University of Calgary

Electronic publication date: 2020 Apr 18

JOURNAL INFORMATION
==============================
NLM Title Abbreviation: Can Med Educ J
Journal ID: Can Med Educ J
NLM Journal ID: 101560935
Journal ID: CMEJ

Canadian Medical Education Journal

EISSN: 1923-1202
Publisher: University of Saskatchewan

ARTICLE INFORMATION
==============================
Subjects: Dp 18–6

Conception et évaluation d'une formation sur les principes de « Choisir avec soin »

Lyne Pitre University of Ottawa

Chantal D'aoust-Bernard University of Ottawa

Anne-Marie Friesen University of Ottawa

David Adjo University of Ottawa

Electronic publication date: 2020 Apr 18

JOURNAL INFORMATION
==============================
NLM Title Abbreviation: Can Med Educ J
Journal ID: Can Med Educ J
NLM Journal ID: 101560935
Journal ID: CMEJ

Canadian Medical Education Journal

EISSN: 1923-1202
Publisher: University of Saskatchewan

ARTICLE INFORMATION
==============================
Subjects: Dp 18–7

Reflexi-Vite: A practical tool for fostering reflexivity in family medicine

Marie-Claude Tremblay Université Laval

Anne Guichard Université Laval

Julien Quinty Université Laval

Christian Rheault Université Laval

Chantal Gravel Université Laval

Marie-Claude Tremblay Université Laval

Electronic publication date: 2020 Apr 18

JOURNAL INFORMATION
==============================
NLM Title Abbreviation: Can Med Educ J
Journal ID: Can Med Educ J
NLM Journal ID: 101560935
Journal ID: CMEJ

Canadian Medical Education Journal

EISSN: 1923-1202
Publisher: University of Saskatchewan

ARTICLE INFORMATION
==============================
Subjects: Dp 18–8

Reflection in healthcare learner : Should it be evaluated and how should it be evaluated?

Cassandra Préfontaine Université de Montréal

Chantal Lemire Université de Sherbrooke

Hélène Corriveau Université de Sherbrooke

Marie-Josée April Université de Sherbrooke

Isabelle Gaboury Université de Sherbrooke

Electronic publication date: 2020 Apr 18

JOURNAL INFORMATION
==============================
NLM Title Abbreviation: Can Med Educ J
Journal ID: Can Med Educ J
NLM Journal ID: 101560935
Journal ID: CMEJ

Canadian Medical Education Journal

EISSN: 1923-1202
Publisher: University of Saskatchewan

ARTICLE INFORMATION
==============================
Subjects: Dp 18–9

Factors That Shape Decision Making Around Resuscitation Preference: A Scoping Review with Implications for Clinical Training

Kristen Bishop Western University

Hasan Hawilo Western University

Ravi Taneja Western University

Mark Goldszmidt Western University

Electronic publication date: 2020 Apr 18

JOURNAL INFORMATION
==============================
NLM Title Abbreviation: Can Med Educ J
Journal ID: Can Med Educ J
NLM Journal ID: 101560935
Journal ID: CMEJ

Canadian Medical Education Journal

EISSN: 1923-1202
Publisher: University of Saskatchewan

ARTICLE INFORMATION
==============================
Subjects: Dp 19–1

Comparison of the English and French versions of the CASPer® Test in a bilingual population

Lemay Genevieve University of Ottawa

Christopher Zou Altus Assessments

John Leddy University of Ottawa

Meghan McConnell University of Ottawa

Patrick Antonacci Altus Assessments

Electronic publication date: 2020 Apr 18

JOURNAL INFORMATION
==============================
NLM Title Abbreviation: Can Med Educ J
Journal ID: Can Med Educ J
NLM Journal ID: 101560935
Journal ID: CMEJ

Canadian Medical Education Journal

EISSN: 1923-1202
Publisher: University of Saskatchewan

ARTICLE INFORMATION
==============================
Subjects: Dp 19–2

Assessing the reflection process: Medical students' perceived consequences

Ann Graillon Université de Sherbrooke

Joanie Poirier Université de Sherbrooke

Kathleen Ouellet Université de Sherbrooke

Valérie Désilets Université de Sherbrooke

Marianne Xhignesse Université de Sherbrooke

Christina St-Onge Université de Sherbrooke

Electronic publication date: 2020 Apr 18

JOURNAL INFORMATION
==============================
NLM Title Abbreviation: Can Med Educ J
Journal ID: Can Med Educ J
NLM Journal ID: 101560935
Journal ID: CMEJ

Canadian Medical Education Journal

EISSN: 1923-1202
Publisher: University of Saskatchewan

ARTICLE INFORMATION
==============================
Subjects: Dp 19–3

The Compatibility Principle: On Philosophies in the Assessment of Clinical Competence

Walter Tavares University of Toronto

Ayelet Kuper University of Toronto

Mahan Kulasegaram University of Toronto

Cynthia Whitehead University of Toronto

Electronic publication date: 2020 Apr 18

JOURNAL INFORMATION
==============================
NLM Title Abbreviation: Can Med Educ J
Journal ID: Can Med Educ J
NLM Journal ID: 101560935
Journal ID: CMEJ

Canadian Medical Education Journal

EISSN: 1923-1202
Publisher: University of Saskatchewan

ARTICLE INFORMATION
==============================
Subjects: Dp 19–4

Defining a set of Palliative Care Entrustable Professional Activities based on Priority Topics and Key Features

Jacqueline Hui University of Calgary

Keith Wycliffe-Jones University of Calgary

Electronic publication date: 2020 Apr 18

JOURNAL INFORMATION
==============================
NLM Title Abbreviation: Can Med Educ J
Journal ID: Can Med Educ J
NLM Journal ID: 101560935
Journal ID: CMEJ

Canadian Medical Education Journal

EISSN: 1923-1202
Publisher: University of Saskatchewan

ARTICLE INFORMATION
==============================
Subjects: Dp 19–5

Specialist assessment activities: The interface of learning, change, and discussion

Jocelyn Lockyer University of Calgary

Jocelyn Lockyer University of Calgary

Craig Campbell The Royal College of Physicians and Surgeons

Shanna DiMillo The Royal College of Physicians and Surgeons

Electronic publication date: 2020 Apr 18

JOURNAL INFORMATION
==============================
NLM Title Abbreviation: Can Med Educ J
Journal ID: Can Med Educ J
NLM Journal ID: 101560935
Journal ID: CMEJ

Canadian Medical Education Journal

EISSN: 1923-1202
Publisher: University of Saskatchewan

ARTICLE INFORMATION
==============================
Subjects: Dp 19–6

Pharmacy students complete formative and summative entrustment assessments (EPAs) to appraise self and peer ability to perform unsupervised in the self-care community workplace

Debra Sibbald University of Toronto

Electronic publication date: 2020 Apr 18

JOURNAL INFORMATION
==============================
NLM Title Abbreviation: Can Med Educ J
Journal ID: Can Med Educ J
NLM Journal ID: 101560935
Journal ID: CMEJ

Canadian Medical Education Journal

EISSN: 1923-1202
Publisher: University of Saskatchewan

ARTICLE INFORMATION
==============================
Subjects: Dp 19–7

Are there gender differences on physicians' reflection and actions taken to improve practice gaps?

Diana C Sanchez-Ramirez University of Manitoba

Hannah Smith University of Manitoba

Dori Rainey University of Manitoba

Electronic publication date: 2020 Apr 18

JOURNAL INFORMATION
==============================
NLM Title Abbreviation: Can Med Educ J
Journal ID: Can Med Educ J
NLM Journal ID: 101560935
Journal ID: CMEJ

Canadian Medical Education Journal

EISSN: 1923-1202
Publisher: University of Saskatchewan

ARTICLE INFORMATION
==============================
Subjects: Dp 19–8

Effect of Station Format on the Psychometric Qualities of Multiple Mini-Interviews

Jean-Sébastien Renaud Université Laval

Martine Bourget Université Laval

Christina St-Onge Université de Sherbrooke

Kevin Eva University of British Columbia

Walter Tavares University of Toronto

Alexis Salvador Loye Université Laval

Jean-Michel Leduc Université de Montréal

Matthew Homer University of Leeds

Electronic publication date: 2020 Apr 18

JOURNAL INFORMATION
==============================
NLM Title Abbreviation: Can Med Educ J
Journal ID: Can Med Educ J
NLM Journal ID: 101560935
Journal ID: CMEJ

Canadian Medical Education Journal

EISSN: 1923-1202
Publisher: University of Saskatchewan

ARTICLE INFORMATION
==============================
Subjects: Dp 19–9

Do high grades prior to MD studies lead to higher academic achievement during the MD curriculum and higher scores on licensing examinations?

Margaret Henri Université de Montréal

Robert Gagnon Université de Montréal

Christian Bourdy Université de Montréal

Jean-Michel Leduc Université de Montréal

Geneviève Grégoire Université de Montréal

Electronic publication date: 2020 Apr 18

JOURNAL INFORMATION
==============================
NLM Title Abbreviation: Can Med Educ J
Journal ID: Can Med Educ J
NLM Journal ID: 101560935
Journal ID: CMEJ

Canadian Medical Education Journal

EISSN: 1923-1202
Publisher: University of Saskatchewan

ARTICLE INFORMATION
==============================
Subjects: Dp 20–1

Practical Approaches to Obesity Management: Interprofessional Care of Patients with a Complex Disease

Boris Zevin Queen’s University

David Barber Queen’s University

Robyn Houlden Queen’s University

Eleftherios Soleas Queen’s University

Lynn Roberts Queen’s University

Richard van Wylick Queen’s University

Katie Evans Queen’s University

Nancy Dalgarno Queen’s University

Electronic publication date: 2020 Apr 18

JOURNAL INFORMATION
==============================
NLM Title Abbreviation: Can Med Educ J
Journal ID: Can Med Educ J
NLM Journal ID: 101560935
Journal ID: CMEJ

Canadian Medical Education Journal

EISSN: 1923-1202
Publisher: University of Saskatchewan

ARTICLE INFORMATION
==============================
Subjects: Dp 20–2

Twelve Tips: Implementing and Sustaining a Successful Interprofessional Simulation Educational Program

Stephen Miller Dalhousie University

Diane MacKenzie Dalhousie University

Gail Creaser Dalhousie University

Kim Sponagle Dalhousie University

Electronic publication date: 2020 Apr 18

JOURNAL INFORMATION
==============================
NLM Title Abbreviation: Can Med Educ J
Journal ID: Can Med Educ J
NLM Journal ID: 101560935
Journal ID: CMEJ

Canadian Medical Education Journal

EISSN: 1923-1202
Publisher: University of Saskatchewan

ARTICLE INFORMATION
==============================
Subjects: Dp 20–3

Interprofessional Collaboration in the Real World - Patient's Medical Home as a foundation for success

Amanda Condon University of Manitoba

Dana Turcotte University of Manitoba

Jessica Clendenan University of Manitoba

Margaret Rauliuk Athabasca University

Electronic publication date: 2020 Apr 18

JOURNAL INFORMATION
==============================
NLM Title Abbreviation: Can Med Educ J
Journal ID: Can Med Educ J
NLM Journal ID: 101560935
Journal ID: CMEJ

Canadian Medical Education Journal

EISSN: 1923-1202
Publisher: University of Saskatchewan

ARTICLE INFORMATION
==============================
Subjects: Dp 20–4

Interprofessional Identity: Are we all on the same plate?

Wendy A Stewart Dalhousie University

Electronic publication date: 2020 Apr 18

JOURNAL INFORMATION
==============================
NLM Title Abbreviation: Can Med Educ J
Journal ID: Can Med Educ J
NLM Journal ID: 101560935
Journal ID: CMEJ

Canadian Medical Education Journal

EISSN: 1923-1202
Publisher: University of Saskatchewan

ARTICLE INFORMATION
==============================
Subjects: Dp 20–5

The Power of Professionalism Award: Fostering Professionalism in an Academic Institution

Ellen M. Friedman Baylor College of Medicine

Larry Laufman Baylor College of Medicine

Joan Friedland Baylor College of Medicine

Anne Gill Baylor College of Medicine

Vandana Shah Baylor Collge of Medicine

Electronic publication date: 2020 Apr 18

JOURNAL INFORMATION
==============================
NLM Title Abbreviation: Can Med Educ J
Journal ID: Can Med Educ J
NLM Journal ID: 101560935
Journal ID: CMEJ

Canadian Medical Education Journal

EISSN: 1923-1202
Publisher: University of Saskatchewan

ARTICLE INFORMATION
==============================
Subjects: Dp 20–6

Promoting the Well-Being and Resilience of Health Professional Learners: Developing and Implementing an Interprofessional Healthy Learning Curriculum

Camila Velez McGill

Deborah Friedman McGill

Nicole-Ann Shery McGill

Electronic publication date: 2020 Apr 18

JOURNAL INFORMATION
==============================
NLM Title Abbreviation: Can Med Educ J
Journal ID: Can Med Educ J
NLM Journal ID: 101560935
Journal ID: CMEJ

Canadian Medical Education Journal

EISSN: 1923-1202
Publisher: University of Saskatchewan

ARTICLE INFORMATION
==============================
Subjects: Dp 20–7

Création d'une formation par concordance interdisciplinaire: présentation du processus et leçons apprises

Rebecca Maftoul Université de Montréal

Vincent Jobin Université de Montréal

Bernard Charlin Université de Montréal

Alexandre Berkesse Centre d'excellence sur le partenariat avec les patients et le public

Annie Descoteaux Université de Montréal

Caroline Lebel Université de Montréal

Marc Rouleau Université de Montréal

Anouck Sénécal Université de Montréal

Electronic publication date: 2020 Apr 18

JOURNAL INFORMATION
==============================
NLM Title Abbreviation: Can Med Educ J
Journal ID: Can Med Educ J
NLM Journal ID: 101560935
Journal ID: CMEJ

Canadian Medical Education Journal

EISSN: 1923-1202
Publisher: University of Saskatchewan

ARTICLE INFORMATION
==============================
Subjects: Dp 21–1

Francisation du curriculum de médecine M.D. de McGill

Tin Ngo-Minh McGill

Gilles Brousseau McGill

Michel Leblanc McGill

Fabien Vadnais McGill

Electronic publication date: 2020 Apr 18

JOURNAL INFORMATION
==============================
NLM Title Abbreviation: Can Med Educ J
Journal ID: Can Med Educ J
NLM Journal ID: 101560935
Journal ID: CMEJ

Canadian Medical Education Journal

EISSN: 1923-1202
Publisher: University of Saskatchewan

ARTICLE INFORMATION
==============================
Subjects: Dp 21–2

Assessing Generalism in Undergraduate Medical Education

Melissa Nutik University of Toronto

Risa Freeman University of Toronto

Nicole Woods University of Toronto

Azadeh Moaveni University of Toronto

Ruby Alvi University of Toronto

James Owen University of Toronto

Jared Gleberzon University of Toronto

Electronic publication date: 2020 Apr 18

JOURNAL INFORMATION
==============================
NLM Title Abbreviation: Can Med Educ J
Journal ID: Can Med Educ J
NLM Journal ID: 101560935
Journal ID: CMEJ

Canadian Medical Education Journal

EISSN: 1923-1202
Publisher: University of Saskatchewan

ARTICLE INFORMATION
==============================
Subjects: Dp 21–3

Evaluation of an on-line transgender health training program

Michael Lee-Poy McMaster University

Electronic publication date: 2020 Apr 18

JOURNAL INFORMATION
==============================
NLM Title Abbreviation: Can Med Educ J
Journal ID: Can Med Educ J
NLM Journal ID: 101560935
Journal ID: CMEJ

Canadian Medical Education Journal

EISSN: 1923-1202
Publisher: University of Saskatchewan

ARTICLE INFORMATION
==============================
Subjects: Dp 21–4

A concerted approach to curriculum mapping by summer students

Sietske Speerstra University of Alberta

Daniel Livy University of Alberta

Hollis Lai University of Alberta

Tracey Hillier University of Alberta

Electronic publication date: 2020 Apr 18

JOURNAL INFORMATION
==============================
NLM Title Abbreviation: Can Med Educ J
Journal ID: Can Med Educ J
NLM Journal ID: 101560935
Journal ID: CMEJ

Canadian Medical Education Journal

EISSN: 1923-1202
Publisher: University of Saskatchewan

ARTICLE INFORMATION
==============================
Subjects: Dp 21–5

Pediatric Developmental Screening Curriculum: Needs Assessment

Cara Dosman University of Alberta

Debbi Andrews University of Alberta

Andrea Davila Cervantes University of Alberta

Qi Guo University of Alberta

Sheila Gallagher University of Alberta

Sabrina Eliason University of Alberta

Carol Hodgson University of Alberta

Electronic publication date: 2020 Apr 18

JOURNAL INFORMATION
==============================
NLM Title Abbreviation: Can Med Educ J
Journal ID: Can Med Educ J
NLM Journal ID: 101560935
Journal ID: CMEJ

Canadian Medical Education Journal

EISSN: 1923-1202
Publisher: University of Saskatchewan

ARTICLE INFORMATION
==============================
Subjects: Dp 21–6

Améliorer la préparation à l'externat : études médicales de premier cycle (émpc), uOttawa

Victoria-Marie Cusson University of Ottawa

Gladys Bruyninx University of Ottawa

Stefan de Laplante University of Ottawa

Electronic publication date: 2020 Apr 18

JOURNAL INFORMATION
==============================
NLM Title Abbreviation: Can Med Educ J
Journal ID: Can Med Educ J
NLM Journal ID: 101560935
Journal ID: CMEJ

Canadian Medical Education Journal

EISSN: 1923-1202
Publisher: University of Saskatchewan

ARTICLE INFORMATION
==============================
Subjects: Dp 21–7

The Impact of PHarmacy Students as Educators (PHASE) and a Simulated Teaching Environment in an Entry-to-Practice PharmD Program at UBC

Fong Chan University of British Columbia

Tony Seet University of British Columbia

Janice Yeung University of British Columbia

Sharon Leung University of British Columbia

Kimberley MacNeil University of British Columbia

Reanne Li University of British Columbia

Justine Saran University of British Columbia

Sandra Jarvis-Selinger University of British Columbia

Electronic publication date: 2020 Apr 18

JOURNAL INFORMATION
==============================
NLM Title Abbreviation: Can Med Educ J
Journal ID: Can Med Educ J
NLM Journal ID: 101560935
Journal ID: CMEJ

Canadian Medical Education Journal

EISSN: 1923-1202
Publisher: University of Saskatchewan

ARTICLE INFORMATION
==============================
Subjects: Dp 21–8

Medical Council of Canada Curricular Gaps: The development and utilization of MCC curricular gap reports

Amanda Stalwick University of Saskatchewan

Regina Taylor-Gjevre University of Saskatchewan

Electronic publication date: 2020 Apr 18

JOURNAL INFORMATION
==============================
NLM Title Abbreviation: Can Med Educ J
Journal ID: Can Med Educ J
NLM Journal ID: 101560935
Journal ID: CMEJ

Canadian Medical Education Journal

EISSN: 1923-1202
Publisher: University of Saskatchewan

ARTICLE INFORMATION
==============================
Subjects: Dp 21–9

A Longitudinal Integrative Resilience Curriculum for Medical Students

Shayna Kulman-Lipsey University of Toronto

Joanne Leo Leo University of Toronto

Samantha Yang University of Toronto

Arshia Javidan University of Toronto

Andrea Levinson University of Toronto

Leslie Nickell University of Toronto

Electronic publication date: 2020 Apr 18

JOURNAL INFORMATION
==============================
NLM Title Abbreviation: Can Med Educ J
Journal ID: Can Med Educ J
NLM Journal ID: 101560935
Journal ID: CMEJ

Canadian Medical Education Journal

EISSN: 1923-1202
Publisher: University of Saskatchewan

ARTICLE INFORMATION
==============================
Subjects: Dp 22–1

Implementation of a Jigsaw Classroom to Teach Pharmacy Students

Colleen Brady University of British Columbia

Michelle Fischer University of British Columbia

Tony Seet University of British Columbia

Electronic publication date: 2020 Apr 18

JOURNAL INFORMATION
==============================
NLM Title Abbreviation: Can Med Educ J
Journal ID: Can Med Educ J
NLM Journal ID: 101560935
Journal ID: CMEJ

Canadian Medical Education Journal

EISSN: 1923-1202
Publisher: University of Saskatchewan

ARTICLE INFORMATION
==============================
Subjects: Dp 22–2

Examining self-regulated learning behaviours of incoming PGY1 residents in two Canadian family medicine programs

Shelley Ross University of Alberta

Alexandra Aquilina University of Alberta

Rob Kiddell University of Victoria

Deena Hamza University of Alberta

Theresa van der Goes University of British Columbia

Cheryl Poth University of Alberta

Shirley Schipper University of Alberta

Electronic publication date: 2020 Apr 18

JOURNAL INFORMATION
==============================
NLM Title Abbreviation: Can Med Educ J
Journal ID: Can Med Educ J
NLM Journal ID: 101560935
Journal ID: CMEJ

Canadian Medical Education Journal

EISSN: 1923-1202
Publisher: University of Saskatchewan

ARTICLE INFORMATION
==============================
Subjects: Dp 22–3

Med Combat Comics - A graphic novel approach to medical education in the armed forces.

Paige Blumer University of British Columbia

Michael Claydon Royal Dragoon Guards, British Army

Katey Murray Engineer Regiment, British Army

Claudia Krebs University of British Columbia

Electronic publication date: 2020 Apr 18

JOURNAL INFORMATION
==============================
NLM Title Abbreviation: Can Med Educ J
Journal ID: Can Med Educ J
NLM Journal ID: 101560935
Journal ID: CMEJ

Canadian Medical Education Journal

EISSN: 1923-1202
Publisher: University of Saskatchewan

ARTICLE INFORMATION
==============================
Subjects: Dp 22–4

A case-based interactive lecture on pathological sonographic findings

Sabine Schneidewind Hannover Medical School

Philip Bintaro Hannover Medical School

Electronic publication date: 2020 Apr 18

JOURNAL INFORMATION
==============================
NLM Title Abbreviation: Can Med Educ J
Journal ID: Can Med Educ J
NLM Journal ID: 101560935
Journal ID: CMEJ

Canadian Medical Education Journal

EISSN: 1923-1202
Publisher: University of Saskatchewan

ARTICLE INFORMATION
==============================
Subjects: Dp 22–5

FAST EVIDENCE: Building a toolkit to optimize patient care

Hadas Haft University of British Columbia

Sarah Tajani University of British Columbia

Bob Bluman University of British Columbia

Karen MacDonell College of Physician and Surgeons of BC

Brenna Lynn University of British Columbia

Electronic publication date: 2020 Apr 18

JOURNAL INFORMATION
==============================
NLM Title Abbreviation: Can Med Educ J
Journal ID: Can Med Educ J
NLM Journal ID: 101560935
Journal ID: CMEJ

Canadian Medical Education Journal

EISSN: 1923-1202
Publisher: University of Saskatchewan

ARTICLE INFORMATION
==============================
Subjects: Dp 22–6

BCPoCUS.ca: A Clinically-Oriented Ultrasound Education Website Interface

Kathryn Young University of British Columbia

Bianca Boicu University of British Columbia

Oron Frenkel St. Paul's Hospital

Electronic publication date: 2020 Apr 18

JOURNAL INFORMATION
==============================
NLM Title Abbreviation: Can Med Educ J
Journal ID: Can Med Educ J
NLM Journal ID: 101560935
Journal ID: CMEJ

Canadian Medical Education Journal

EISSN: 1923-1202
Publisher: University of Saskatchewan

ARTICLE INFORMATION
==============================
Subjects: Dp 22–7

Experiential Nursing Rounds: An opportunity for graduate nurses to process challenges, develop coping strategies, and experience social support within the academic curriculum

Camila Velez McGill

Nicole-Ann Shery McGill

Deborah Friedman McGill

Electronic publication date: 2020 Apr 18

JOURNAL INFORMATION
==============================
NLM Title Abbreviation: Can Med Educ J
Journal ID: Can Med Educ J
NLM Journal ID: 101560935
Journal ID: CMEJ

Canadian Medical Education Journal

EISSN: 1923-1202
Publisher: University of Saskatchewan

ARTICLE INFORMATION
==============================
Subjects: Dp 22–8

Assessing mistreatment among medical students at different stages of undergraduate education

Richard Pittini University of Toronto

Yuxin Tu University of Toronto

Lisa Robinson University of Toronto

Glenys Babcock University of Toronto

Mariela Ruetalo University of Toronto

Electronic publication date: 2020 Apr 18

JOURNAL INFORMATION
==============================
NLM Title Abbreviation: Can Med Educ J
Journal ID: Can Med Educ J
NLM Journal ID: 101560935
Journal ID: CMEJ

Canadian Medical Education Journal

EISSN: 1923-1202
Publisher: University of Saskatchewan

ARTICLE INFORMATION
==============================
Subjects: Dp 23–1

Project-Managing the Poetry of Medicine: Structure and Meaning in an Accreditation and Quality Improvement Unit

Fernanda Claudio McGill

Patricia Wade McGill

Electronic publication date: 2020 Apr 18

JOURNAL INFORMATION
==============================
NLM Title Abbreviation: Can Med Educ J
Journal ID: Can Med Educ J
NLM Journal ID: 101560935
Journal ID: CMEJ

Canadian Medical Education Journal

EISSN: 1923-1202
Publisher: University of Saskatchewan

ARTICLE INFORMATION
==============================
Subjects: Dp 23–2

Effective speakers in Continuing Medical Education

Diana C Sanchez-Ramirez University of Manitoba

Christine Polimeni University of Manitoba

Electronic publication date: 2020 Apr 18

JOURNAL INFORMATION
==============================
NLM Title Abbreviation: Can Med Educ J
Journal ID: Can Med Educ J
NLM Journal ID: 101560935
Journal ID: CMEJ

Canadian Medical Education Journal

EISSN: 1923-1202
Publisher: University of Saskatchewan

ARTICLE INFORMATION
==============================
Subjects: Dp 23–3

Are We Ready For What's Coming? Scholarship, Leadership and Continuing Professional Development

Morag Paton University of Toronto

Paula Rowland University of Toronto

Walter Tavares University of Toronto

Suzan Schneeweiss University of Toronto

Shiphra Ginsburg University of Toronto

Electronic publication date: 2020 Apr 18

JOURNAL INFORMATION
==============================
NLM Title Abbreviation: Can Med Educ J
Journal ID: Can Med Educ J
NLM Journal ID: 101560935
Journal ID: CMEJ

Canadian Medical Education Journal

EISSN: 1923-1202
Publisher: University of Saskatchewan

ARTICLE INFORMATION
==============================
Subjects: Dp 23–4

The changing landscape of Continuing Professional Development/Continuing Medical Education (CPD/CME) in Canada: A scoping review.

Francesca Luconi McGill

Elizabeth Wooster University of Toronto

Morag Paton University of Toronto

Meron Teferra McGill

Andrea Quaiattini McGill

Suzan Schneeweiss University of Toronto

Electronic publication date: 2020 Apr 18

JOURNAL INFORMATION
==============================
NLM Title Abbreviation: Can Med Educ J
Journal ID: Can Med Educ J
NLM Journal ID: 101560935
Journal ID: CMEJ

Canadian Medical Education Journal

EISSN: 1923-1202
Publisher: University of Saskatchewan

ARTICLE INFORMATION
==============================
Subjects: Dp 23–5

Auditing Rounds in a Distributed Medical Education Environment: An Innovative Approach

Clare Cook Northern Ontario School of Medicine

Lisa Kokanie Northern Ontario School of Medicine

Julie Colquhoun Northern Ontario School of Medicine

Electronic publication date: 2020 Apr 18

JOURNAL INFORMATION
==============================
NLM Title Abbreviation: Can Med Educ J
Journal ID: Can Med Educ J
NLM Journal ID: 101560935
Journal ID: CMEJ

Canadian Medical Education Journal

EISSN: 1923-1202
Publisher: University of Saskatchewan

ARTICLE INFORMATION
==============================
Subjects: Dp 23–6

A continuous quality improvement model for person-centered clinical education.

Susan Cauti University of Alberta

Heather Gaunt University of Alberta

Kari Osmar University of Alberta

Electronic publication date: 2020 Apr 18

JOURNAL INFORMATION
==============================
NLM Title Abbreviation: Can Med Educ J
Journal ID: Can Med Educ J
NLM Journal ID: 101560935
Journal ID: CMEJ

Canadian Medical Education Journal

EISSN: 1923-1202
Publisher: University of Saskatchewan

ARTICLE INFORMATION
==============================
Subjects: Dp 23–7

The use of theory in quality improvement and patient safety education: a scoping review

Joanne Goldman University of Toronto

Brian Wong University of Toronto

Lisha Lo University of Toronto

Ayelet Kuper University of Toronto

Anne Smeraglio Portland Veterans Hospital and Oregon Health & Science University

Electronic publication date: 2020 Apr 18

JOURNAL INFORMATION
==============================
NLM Title Abbreviation: Can Med Educ J
Journal ID: Can Med Educ J
NLM Journal ID: 101560935
Journal ID: CMEJ

Canadian Medical Education Journal

EISSN: 1923-1202
Publisher: University of Saskatchewan

ARTICLE INFORMATION
==============================
Subjects: Dp 23–8

Rural Medicine eMentoring BC (RMeMBC)

Takaia Larsen Selkirk College

Electronic publication date: 2020 Apr 18

JOURNAL INFORMATION
==============================
NLM Title Abbreviation: Can Med Educ J
Journal ID: Can Med Educ J
NLM Journal ID: 101560935
Journal ID: CMEJ

Canadian Medical Education Journal

EISSN: 1923-1202
Publisher: University of Saskatchewan

ARTICLE INFORMATION
==============================
Subjects: Dp 24–1

Class Years Have Reputations As Hawks or Doves, But Are They?

Kevin Busche University of Calgary

Mike Paget University of Calgary

Wayne Woloschuk University of Calgary

Electronic publication date: 2020 Apr 18

JOURNAL INFORMATION
==============================
NLM Title Abbreviation: Can Med Educ J
Journal ID: Can Med Educ J
NLM Journal ID: 101560935
Journal ID: CMEJ

Canadian Medical Education Journal

EISSN: 1923-1202
Publisher: University of Saskatchewan

ARTICLE INFORMATION
==============================
Subjects: Dp 24–2

Non-Critical Care Residents Exposure to Clinical and Educational Activities During ICU Rotations: A multicentered, observational study in 3 Academic ICUs

Dominique Piquette University of Toronto

Briseida Mema University of Toronto

Christie Lee University of Toronto

Alberto Goffi University of Toronto

Catharine Walsh University of Toronto

Ryan Brydges University of Toronto

Electronic publication date: 2020 Apr 18

JOURNAL INFORMATION
==============================
NLM Title Abbreviation: Can Med Educ J
Journal ID: Can Med Educ J
NLM Journal ID: 101560935
Journal ID: CMEJ

Canadian Medical Education Journal

EISSN: 1923-1202
Publisher: University of Saskatchewan

ARTICLE INFORMATION
==============================
Subjects: Dp 24–3

The clinical teaching practicum: an important learning methodology for instructional development

Debaroti Mullick Borschel University of Tennnessee

Umberin Najeeb University of Toronto

Danny Panisko University of Toronto

Electronic publication date: 2020 Apr 18

JOURNAL INFORMATION
==============================
NLM Title Abbreviation: Can Med Educ J
Journal ID: Can Med Educ J
NLM Journal ID: 101560935
Journal ID: CMEJ

Canadian Medical Education Journal

EISSN: 1923-1202
Publisher: University of Saskatchewan

ARTICLE INFORMATION
==============================
Subjects: Dp 24–4

University of Toronto (U. of T.) Postgraduate Medical Education: Competency-Based Medical Education Faculty Development Survey

Giovanna Sirianni University of Toronto

Susan Glover-Takahashi University of Toronto

Electronic publication date: 2020 Apr 18

JOURNAL INFORMATION
==============================
NLM Title Abbreviation: Can Med Educ J
Journal ID: Can Med Educ J
NLM Journal ID: 101560935
Journal ID: CMEJ

Canadian Medical Education Journal

EISSN: 1923-1202
Publisher: University of Saskatchewan

ARTICLE INFORMATION
==============================
Subjects: Dp 24–5

PANPro: a comprehensive one-year Faculty development program for new teachers

Lise Cusson Université de Montréal

Suzanne Laurin Université de Montréal

Electronic publication date: 2020 Apr 18

JOURNAL INFORMATION
==============================
NLM Title Abbreviation: Can Med Educ J
Journal ID: Can Med Educ J
NLM Journal ID: 101560935
Journal ID: CMEJ

Canadian Medical Education Journal

EISSN: 1923-1202
Publisher: University of Saskatchewan

ARTICLE INFORMATION
==============================
Subjects: Dp 24–6

Which components of the INTAPT Faculty Development course do learners identify as impactful to their learning

Judith Peranson University of Toronto

Helen Batty University of Toronto

Ancy Jacob University of Toronto

Abbas Ghavam-Rassoul University of Toronto

Electronic publication date: 2020 Apr 18

JOURNAL INFORMATION
==============================
NLM Title Abbreviation: Can Med Educ J
Journal ID: Can Med Educ J
NLM Journal ID: 101560935
Journal ID: CMEJ

Canadian Medical Education Journal

EISSN: 1923-1202
Publisher: University of Saskatchewan

ARTICLE INFORMATION
==============================
Subjects: Dp 24–7

Faculty development to support curriculum renewal: An aligned approach

Jana Lazor University of Toronto

Jennifer Bell University of Toronto

Lori Innes University of Toronto

Electronic publication date: 2020 Apr 18

JOURNAL INFORMATION
==============================
NLM Title Abbreviation: Can Med Educ J
Journal ID: Can Med Educ J
NLM Journal ID: 101560935
Journal ID: CMEJ

Canadian Medical Education Journal

EISSN: 1923-1202
Publisher: University of Saskatchewan

ARTICLE INFORMATION
==============================
Subjects: Dp 25–1

The Early Introduction of Ultrasound in Undergraduate Medicine

Eleanor Good University of British Columbia

Pete Tonseth University of British Columbia

Anthony Sturgeon University of British Columbia

Angelina Marinkovic University of British Columbia

Alasdair Nazerali-Maitland University of British Columbia

Samantha Stasiuk University of British Columbia

Electronic publication date: 2020 Apr 18

JOURNAL INFORMATION
==============================
NLM Title Abbreviation: Can Med Educ J
Journal ID: Can Med Educ J
NLM Journal ID: 101560935
Journal ID: CMEJ

Canadian Medical Education Journal

EISSN: 1923-1202
Publisher: University of Saskatchewan

ARTICLE INFORMATION
==============================
Subjects: Dp 25–2

Using Outcome Harvesting to Assess CBME Implementation at Queen's University

Jennifer Railer Queen’s University

Denise Stockley Queen’s University

Leslie Flynn Queen’s University

Amber Hastings-Truelove Queen’s University

Alicia Hussain Queen’s University

Electronic publication date: 2020 Apr 18

JOURNAL INFORMATION
==============================
NLM Title Abbreviation: Can Med Educ J
Journal ID: Can Med Educ J
NLM Journal ID: 101560935
Journal ID: CMEJ

Canadian Medical Education Journal

EISSN: 1923-1202
Publisher: University of Saskatchewan

ARTICLE INFORMATION
==============================
Subjects: Dp 25–3

Un service de consultation pédagogique pour les cliniciens enseignants

Suzanne Laurin Université de Montréal

Electronic publication date: 2020 Apr 18

JOURNAL INFORMATION
==============================
NLM Title Abbreviation: Can Med Educ J
Journal ID: Can Med Educ J
NLM Journal ID: 101560935
Journal ID: CMEJ

Canadian Medical Education Journal

EISSN: 1923-1202
Publisher: University of Saskatchewan

ARTICLE INFORMATION
==============================
Subjects: Dp 25–4

General Internal Medicine Fellows as Teachers in the Ambulatory Clinic

Cary Cuncic University of British Columbia

James Tessaro University of British Columbia

Harp Nagi University of British Columbia

Allison Nakanishi University of British Columbia

Aman Nijjar University of British Columbia

Electronic publication date: 2020 Apr 18

JOURNAL INFORMATION
==============================
NLM Title Abbreviation: Can Med Educ J
Journal ID: Can Med Educ J
NLM Journal ID: 101560935
Journal ID: CMEJ

Canadian Medical Education Journal

EISSN: 1923-1202
Publisher: University of Saskatchewan

ARTICLE INFORMATION
==============================
Subjects: Dp 25–5

The Impact of Urban-based Family Medicine Postgraduate Rotations on Rural Preceptor/Teachers (IMPORT)

Douglas Myhre University of Calgary

Rebecca Malhi University of Calgary

Jodie Ornstein University of Calgary

Molly Whalen-Browne University of Calgary

Electronic publication date: 2020 Apr 18

JOURNAL INFORMATION
==============================
NLM Title Abbreviation: Can Med Educ J
Journal ID: Can Med Educ J
NLM Journal ID: 101560935
Journal ID: CMEJ

Canadian Medical Education Journal

EISSN: 1923-1202
Publisher: University of Saskatchewan

ARTICLE INFORMATION
==============================
Subjects: Dp 25–6

Clinical Learning Experiences and Resident Gender

Sonya Lee University of Calgary

Sudha Koppula University of Alberta

Stephen Mintsioulis University of Calgary

Maureen Topps University of Calgary

Sarah Jacobs University of Calgary

Electronic publication date: 2020 Apr 18

JOURNAL INFORMATION
==============================
NLM Title Abbreviation: Can Med Educ J
Journal ID: Can Med Educ J
NLM Journal ID: 101560935
Journal ID: CMEJ

Canadian Medical Education Journal

EISSN: 1923-1202
Publisher: University of Saskatchewan

ARTICLE INFORMATION
==============================
Subjects: Dp 25–7

Addictions 101 for Primary Care: Piloting an Addictions Curriculum with Family Practice Residents

Jennifer Wyman University of Toronto

Ethan Cohen University of Toronto

Anne Wideman University of Toronto

Andrea David University of Toronto

Joyce Nyhof-Young University of Toronto

Mira Shuman University of Toronto

Electronic publication date: 2020 Apr 18

JOURNAL INFORMATION
==============================
NLM Title Abbreviation: Can Med Educ J
Journal ID: Can Med Educ J
NLM Journal ID: 101560935
Journal ID: CMEJ

Canadian Medical Education Journal

EISSN: 1923-1202
Publisher: University of Saskatchewan

ARTICLE INFORMATION
==============================
Subjects: Dp 25–8

Reflection and Renewal: A Critical Review of the Teaching Residents to Teach Program for Family Medicine Residents at the University of Toronto

Heather Zimcik University of Toronto

Marcus Law University of Toronto

Jennifer McCabe University of Toronto

Stuart Murdoch University of Toronto

Azadeh Moaveni University of Toronto

Risa Freeman University of Toronto

Electronic publication date: 2020 Apr 18

JOURNAL INFORMATION
==============================
NLM Title Abbreviation: Can Med Educ J
Journal ID: Can Med Educ J
NLM Journal ID: 101560935
Journal ID: CMEJ

Canadian Medical Education Journal

EISSN: 1923-1202
Publisher: University of Saskatchewan

ARTICLE INFORMATION
==============================
Subjects: Dp 25–9

Representation of Women Presenters at Psychiatry Grand Rounds

Mandy Esliger Dalhousie University

Lara Hazelton Dalhousie University

Heather Milliken Dalhousie University

Kim Good Dalhousie University

Aditya Nidumolu Dalhousie University

Electronic publication date: 2020 Apr 18

JOURNAL INFORMATION
==============================
NLM Title Abbreviation: Can Med Educ J
Journal ID: Can Med Educ J
NLM Journal ID: 101560935
Journal ID: CMEJ

Canadian Medical Education Journal

EISSN: 1923-1202
Publisher: University of Saskatchewan

ARTICLE INFORMATION
==============================
Subjects: Dp 26–1

Mapping Clinical Relevance Network

Kuo-Hsing Kuo University of British Columbia

Joyce Leo Department of Laboratory Medicine, Royal Jubilee Hospital

Electronic publication date: 2020 Apr 18

JOURNAL INFORMATION
==============================
NLM Title Abbreviation: Can Med Educ J
Journal ID: Can Med Educ J
NLM Journal ID: 101560935
Journal ID: CMEJ

Canadian Medical Education Journal

EISSN: 1923-1202
Publisher: University of Saskatchewan

ARTICLE INFORMATION
==============================
Subjects: Dp 26–2

Sipping from the firehose: organizational readiness for big data in learning analytics

David Topps University of Calgary

Electronic publication date: 2020 Apr 18

JOURNAL INFORMATION
==============================
NLM Title Abbreviation: Can Med Educ J
Journal ID: Can Med Educ J
NLM Journal ID: 101560935
Journal ID: CMEJ

Canadian Medical Education Journal

EISSN: 1923-1202
Publisher: University of Saskatchewan

ARTICLE INFORMATION
==============================
Subjects: Dp 26–3

How can the experiences of stakeholders with doctors inform medical selection and education? An interpretive phenomenological study.

Marise Lombard Griffith University

Marise Lombard Griffith University

Electronic publication date: 2020 Apr 18

JOURNAL INFORMATION
==============================
NLM Title Abbreviation: Can Med Educ J
Journal ID: Can Med Educ J
NLM Journal ID: 101560935
Journal ID: CMEJ

Canadian Medical Education Journal

EISSN: 1923-1202
Publisher: University of Saskatchewan

ARTICLE INFORMATION
==============================
Subjects: Dp 26–4

Elicit 'Rich Data' with 'Rich Pictures': An example exploring palliative care learning in rural Canada

King Keely Northern Ontario School of Medicine

Frances Kilbertus Northern Ontario School of Medicine

Susan Robinson Northern Ontario School of Medicine

Sayra Cristancho Western University

Sarah Burm Dalhousie University

Electronic publication date: 2020 Apr 18

JOURNAL INFORMATION
==============================
NLM Title Abbreviation: Can Med Educ J
Journal ID: Can Med Educ J
NLM Journal ID: 101560935
Journal ID: CMEJ

Canadian Medical Education Journal

EISSN: 1923-1202
Publisher: University of Saskatchewan

ARTICLE INFORMATION
==============================
Subjects: Dp 26–5

There's got to be a better way: Institutional Ethnography of intrapartum practices on a Labour & Delivery unit

Stella Ng St. Michael's Hospital, Unity Health Toronto

Lori Nemoy St. Michael's Hospital, Unity Health Toronto

Douglas Campbell St. Michael's Hospital, Unity Health Toronto

Filomena Meffe St. Michael's Hospital, Unity Health Toronto

Linda Moscovitch St. Michael's Hospital, Unity Health Toronto

Catherine Bishop St. Michael's Hospital, Unity Health Toronto

Sabina Fella St. Michael's Hospital, Unity Health Toronto

Nirmala Chandrasekaran St. Michael's Hospital, Unity Health Toronto

Ryan Brydges St. Michael's Hospital, Unity Health Toronto

Electronic publication date: 2020 Apr 18

JOURNAL INFORMATION
==============================
NLM Title Abbreviation: Can Med Educ J
Journal ID: Can Med Educ J
NLM Journal ID: 101560935
Journal ID: CMEJ

Canadian Medical Education Journal

EISSN: 1923-1202
Publisher: University of Saskatchewan

ARTICLE INFORMATION
==============================
Subjects: Dp 26–6

Analyser les interactions patient/médecin lors de téléconsultations : comprendre la pratique pour mieux former

Sylvie Grosjean University of Ottawa

Luc Bonneville University of Ottawa

Richard Waldolf Institut du Savoir Montfort / Université d'Ottawa

Isaac Nahon-Serfaty University of Ottawa

Electronic publication date: 2020 Apr 18

JOURNAL INFORMATION
==============================
NLM Title Abbreviation: Can Med Educ J
Journal ID: Can Med Educ J
NLM Journal ID: 101560935
Journal ID: CMEJ

Canadian Medical Education Journal

EISSN: 1923-1202
Publisher: University of Saskatchewan

ARTICLE INFORMATION
==============================
Subjects: Dp 26–7

Technology and clinician-learner interaction: How is the introduction of a new electronic health record expected to affect educational practice?

Julianna Caon University of British Columbia

Kevin Eva University of British Columbia

Electronic publication date: 2020 Apr 18

JOURNAL INFORMATION
==============================
NLM Title Abbreviation: Can Med Educ J
Journal ID: Can Med Educ J
NLM Journal ID: 101560935
Journal ID: CMEJ

Canadian Medical Education Journal

EISSN: 1923-1202
Publisher: University of Saskatchewan

ARTICLE INFORMATION
==============================
Subjects: Dp 26–8

Use of Simulation in Canadian Undergraduate Medical Schools

Stephen Miller Dalhousie University

Stuart McAdam Dalhousie University

Tasha Kulai Dalhousie University

Babar Haroon Dalhousie University

Electronic publication date: 2020 Apr 18

JOURNAL INFORMATION
==============================
NLM Title Abbreviation: Can Med Educ J
Journal ID: Can Med Educ J
NLM Journal ID: 101560935
Journal ID: CMEJ

Canadian Medical Education Journal

EISSN: 1923-1202
Publisher: University of Saskatchewan

ARTICLE INFORMATION
==============================
Subjects: Dp 27–1

Longitudinal Evaluation of Peer Assessment of Professional Competence in Undergraduate Medical Education

Nicholas Fairbridge Memorial University of Newfoundland

Vernon Curran Memorial University of Newfoundland

Diana Deacon Memorial University of Newfoundland

Electronic publication date: 2020 Apr 18

JOURNAL INFORMATION
==============================
NLM Title Abbreviation: Can Med Educ J
Journal ID: Can Med Educ J
NLM Journal ID: 101560935
Journal ID: CMEJ

Canadian Medical Education Journal

EISSN: 1923-1202
Publisher: University of Saskatchewan

ARTICLE INFORMATION
==============================
Subjects: Dp 27–2

Teaching Professional Practice: “Escape Room” Style

Karen Sauve University of British Columbia

Alison Greig University of British Columbia

Sue Murphy University of British Columbia

Riley Louie University of British Columbia

Joseph Anthony University of British Columbia

Electronic publication date: 2020 Apr 18

JOURNAL INFORMATION
==============================
NLM Title Abbreviation: Can Med Educ J
Journal ID: Can Med Educ J
NLM Journal ID: 101560935
Journal ID: CMEJ

Canadian Medical Education Journal

EISSN: 1923-1202
Publisher: University of Saskatchewan

ARTICLE INFORMATION
==============================
Subjects: Dp 27–3

Tacos with Teachers...Increasing the Connection Between Learners and Faculty

Ellen M. Friedman Baylor College of Medicine

Toi Harris Baylor College of Medicine

Erik Malmberg Baylor College of Medicine

Electronic publication date: 2020 Apr 18

JOURNAL INFORMATION
==============================
NLM Title Abbreviation: Can Med Educ J
Journal ID: Can Med Educ J
NLM Journal ID: 101560935
Journal ID: CMEJ

Canadian Medical Education Journal

EISSN: 1923-1202
Publisher: University of Saskatchewan

ARTICLE INFORMATION
==============================
Subjects: Dp 27–4

It's not about being 'nice': A Serious Illness Communication Curriculum for Cardiology Residents

Katie Marchington University of Toronto

Warren Lewin University of Toronto

Electronic publication date: 2020 Apr 18

JOURNAL INFORMATION
==============================
NLM Title Abbreviation: Can Med Educ J
Journal ID: Can Med Educ J
NLM Journal ID: 101560935
Journal ID: CMEJ

Canadian Medical Education Journal

EISSN: 1923-1202
Publisher: University of Saskatchewan

ARTICLE INFORMATION
==============================
Subjects: Dp 27–5

Factors influencing credibility of instructors in a faculty development program about leadership

Lara Hazelton Dalhousie University

Susan Love Dalhousie University

Lisa Bonang Dalhousie University

Electronic publication date: 2020 Apr 18

JOURNAL INFORMATION
==============================
NLM Title Abbreviation: Can Med Educ J
Journal ID: Can Med Educ J
NLM Journal ID: 101560935
Journal ID: CMEJ

Canadian Medical Education Journal

EISSN: 1923-1202
Publisher: University of Saskatchewan

ARTICLE INFORMATION
==============================
Subjects: Dp 27–6

Exploring Perceptions of Physician Leadership and Character Development Among Doctors, Nurses and Allied Health Members

Jacqueline Torti Western University

Ali Inayat Western University

Hamza Inayat Western University

Wael Haddara Western University

Nabil Sultan Western University

Electronic publication date: 2020 Apr 18

JOURNAL INFORMATION
==============================
NLM Title Abbreviation: Can Med Educ J
Journal ID: Can Med Educ J
NLM Journal ID: 101560935
Journal ID: CMEJ

Canadian Medical Education Journal

EISSN: 1923-1202
Publisher: University of Saskatchewan

ARTICLE INFORMATION
==============================
Subjects: Dp 27–7

Le profil du professionnel de la santé socialement responsable: le point de vue des citoyens

Emmanuelle Careau Université Laval

Marie-Claire Bérubé Université Laval

Émélie Provost Université Laval

Julien Poitras Université Laval

Electronic publication date: 2020 Apr 18

JOURNAL INFORMATION
==============================
NLM Title Abbreviation: Can Med Educ J
Journal ID: Can Med Educ J
NLM Journal ID: 101560935
Journal ID: CMEJ

Canadian Medical Education Journal

EISSN: 1923-1202
Publisher: University of Saskatchewan

ARTICLE INFORMATION
==============================
Subjects: Dp 27–8

An Online Training Program for Clinicians using Telemedicine: From Research to Design

Sylvie Grosjean University of Ottawa

Richard Waldolf Institut du Savoir Montfort

Luc Bonneville University of Ottawa

Judith Boileau Institut du Savoir Montfort

Isaac Nahon-Serfaty University of Ottawa

Maria Cherba Université de Montréal

Electronic publication date: 2020 Apr 18

JOURNAL INFORMATION
==============================
NLM Title Abbreviation: Can Med Educ J
Journal ID: Can Med Educ J
NLM Journal ID: 101560935
Journal ID: CMEJ

Canadian Medical Education Journal

EISSN: 1923-1202
Publisher: University of Saskatchewan

ARTICLE INFORMATION
==============================
Subjects: Dp 27–9

A Workshop to Advance Communication and Primary Palliative Care Skills of 4th Year Medical Students

Tamara Vesel Tufts Medical Center

Grace Wang Tufts medical center

Maria Blanco Tufts University School of Medicine

Emma Ernst Tufts University School of Medicine

Electronic publication date: 2020 Apr 18

JOURNAL INFORMATION
==============================
NLM Title Abbreviation: Can Med Educ J
Journal ID: Can Med Educ J
NLM Journal ID: 101560935
Journal ID: CMEJ

Canadian Medical Education Journal

EISSN: 1923-1202
Publisher: University of Saskatchewan

ARTICLE INFORMATION
==============================
Subjects: Dp 28–1

UBC Indigenous Patient Voice: An Innovative Standardized Patient Session to Develop Indigenous Cultural Safety and to Facilitate Indigenous Instructor Recruitment and Retention

Leah Walker University of British Columbia

Rebecca Howse University of British Columbia

Rebekah Eatmon University of British Columbia

Electronic publication date: 2020 Apr 18

JOURNAL INFORMATION
==============================
NLM Title Abbreviation: Can Med Educ J
Journal ID: Can Med Educ J
NLM Journal ID: 101560935
Journal ID: CMEJ

Canadian Medical Education Journal

EISSN: 1923-1202
Publisher: University of Saskatchewan

ARTICLE INFORMATION
==============================
Subjects: Dp 28–2

Learning by listening to patients: Infusing Patient Voice into Online Education

Yan Chow University of British Columbia

Kate Campbell University of British Columbia

Claudia Hopkins University of British Columbia

Andrea Keesey University of British Columbia

Brenna Lynn University of British Columbia

Electronic publication date: 2020 Apr 18

JOURNAL INFORMATION
==============================
NLM Title Abbreviation: Can Med Educ J
Journal ID: Can Med Educ J
NLM Journal ID: 101560935
Journal ID: CMEJ

Canadian Medical Education Journal

EISSN: 1923-1202
Publisher: University of Saskatchewan

ARTICLE INFORMATION
==============================
Subjects: Dp 28–3

Patients impliqués en éducation des professionnels de santé (PIEPS) : des besoins de formations communes et spécifiques pour chaque groupe.

Isabelle Burnier University of Ottawa

Salomon Fotsing University of Ottawa

Marie Hélène Chomienne University of Ottawa

Diane Bouchard-Lamothe University of Ottawa

Electronic publication date: 2020 Apr 18

JOURNAL INFORMATION
==============================
NLM Title Abbreviation: Can Med Educ J
Journal ID: Can Med Educ J
NLM Journal ID: 101560935
Journal ID: CMEJ

Canadian Medical Education Journal

EISSN: 1923-1202
Publisher: University of Saskatchewan

ARTICLE INFORMATION
==============================
Subjects: Dp 28–4

How to design courses in partnership with patients

Mathieu Jackson Université de Montréal

Annie Descoteaux Université de Montréal

Louise Nicaise Université de Montréal

Philippe Karazivan Université de Montréal

Marie-Pierre Codsi Université de Montréal

Clara Dallaire Université de Montréal

Electronic publication date: 2020 Apr 18

JOURNAL INFORMATION
==============================
NLM Title Abbreviation: Can Med Educ J
Journal ID: Can Med Educ J
NLM Journal ID: 101560935
Journal ID: CMEJ

Canadian Medical Education Journal

EISSN: 1923-1202
Publisher: University of Saskatchewan

ARTICLE INFORMATION
==============================
Subjects: Dp 28–5

Scoping review of Refugee Health Curriculum in Undergraduate Medical Education (UME)

Andrea Davila-Cervantes University of Alberta

Marghalara Rashid University of Alberta

Helly Goez University of Alberta

Electronic publication date: 2020 Apr 18

JOURNAL INFORMATION
==============================
NLM Title Abbreviation: Can Med Educ J
Journal ID: Can Med Educ J
NLM Journal ID: 101560935
Journal ID: CMEJ

Canadian Medical Education Journal

EISSN: 1923-1202
Publisher: University of Saskatchewan

ARTICLE INFORMATION
==============================
Subjects: Dp 28–6

Community Perspectives: Results from community partners' formal review of the Service Learning program for undergraduate medical students

Karen Cook University of Manitoba

Chelsea Jalloh University of Manitoba

Roger Berrington Canu - Service Learning Site

Nina Condo Elmwood Community Resource Centre - Service Learning Community Partner

Felicien Rubayita Welcome Place - Service Learning Site

Ian Whetter University of Manitoba

Electronic publication date: 2020 Apr 18

JOURNAL INFORMATION
==============================
NLM Title Abbreviation: Can Med Educ J
Journal ID: Can Med Educ J
NLM Journal ID: 101560935
Journal ID: CMEJ

Canadian Medical Education Journal

EISSN: 1923-1202
Publisher: University of Saskatchewan

ARTICLE INFORMATION
==============================
Subjects: Dp 28–7

Rural Longitudinal Integrated Clerkship: An answer to increasing numbers of graduates choosing family medicine & rural practice locations

Jill Konkin University of Alberta

Daniel Lemoine University of Alberta

Liam Rourke University of Alberta

Darren Nichols University of Alberta

Electronic publication date: 2020 Apr 18

JOURNAL INFORMATION
==============================
NLM Title Abbreviation: Can Med Educ J
Journal ID: Can Med Educ J
NLM Journal ID: 101560935
Journal ID: CMEJ

Canadian Medical Education Journal

EISSN: 1923-1202
Publisher: University of Saskatchewan

ARTICLE INFORMATION
==============================
Subjects: Dp 28–8

The Senior Citizen Partnership Program (SCPP): a win-win for both medical students and the elderly generation

Ute Hauck Curtin Medical School

Electronic publication date: 2020 Apr 18

JOURNAL INFORMATION
==============================
NLM Title Abbreviation: Can Med Educ J
Journal ID: Can Med Educ J
NLM Journal ID: 101560935
Journal ID: CMEJ

Canadian Medical Education Journal

EISSN: 1923-1202
Publisher: University of Saskatchewan

ARTICLE INFORMATION
==============================
Subjects: Dp 29–1

Scholarly Activity as a Selection Criteria in the Canadian Residency Matching Service (CaRMS): a review of published criteria by internal medicine, family medicine and pediatrics programs

Amanda Bell McMaster University

Karl Stobbe McMaster University

Larry W. Chambers McMaster University

Vesa Basha McMaster University

Jessie Brazier McMaster University

Delia Dragomir McMaster University

Meghan Glibbery McMaster University

Hannah Kearney McMaster University

Alison Knapp McMaster University

Daniel Levin McMaster University

Dyon Tucker McMaster University

Seddiq Weera McMaster University

Jorin Lukings McMaster University

Electronic publication date: 2020 Apr 18

JOURNAL INFORMATION
==============================
NLM Title Abbreviation: Can Med Educ J
Journal ID: Can Med Educ J
NLM Journal ID: 101560935
Journal ID: CMEJ

Canadian Medical Education Journal

EISSN: 1923-1202
Publisher: University of Saskatchewan

ARTICLE INFORMATION
==============================
Subjects: Dp 29–2

Histology Milestones and Exit Competencies for Canadian Undergraduate Medical Education

Karen Pinder University of British Columbia

Jason Ford Sidra Medicine

Electronic publication date: 2020 Apr 18

JOURNAL INFORMATION
==============================
NLM Title Abbreviation: Can Med Educ J
Journal ID: Can Med Educ J
NLM Journal ID: 101560935
Journal ID: CMEJ

Canadian Medical Education Journal

EISSN: 1923-1202
Publisher: University of Saskatchewan

ARTICLE INFORMATION
==============================
Subjects: Dp 29–3

Distributed Specialty Medical Education in Canada: A Survey

Charles Penner University of Manitoba

Electronic publication date: 2020 Apr 18

JOURNAL INFORMATION
==============================
NLM Title Abbreviation: Can Med Educ J
Journal ID: Can Med Educ J
NLM Journal ID: 101560935
Journal ID: CMEJ

Canadian Medical Education Journal

EISSN: 1923-1202
Publisher: University of Saskatchewan

ARTICLE INFORMATION
==============================
Subjects: Dp 29–4

L'évaluation des impacts de l'enseignement médical décentralisé, perception des acteurs.

Jean Ouellet Université Laval

Elise Martel Université Laval

Sébastien Turgeon Université Laval

Alexandra Dubé-Louber Université Laval

Julie Fortin Université Laval

Raymond Thibodeau Université Laval

Mathieu Pelletier Université Laval

Diane Comeau Université Laval

Claudine Parent Université Laval

Electronic publication date: 2020 Apr 18

JOURNAL INFORMATION
==============================
NLM Title Abbreviation: Can Med Educ J
Journal ID: Can Med Educ J
NLM Journal ID: 101560935
Journal ID: CMEJ

Canadian Medical Education Journal

EISSN: 1923-1202
Publisher: University of Saskatchewan

ARTICLE INFORMATION
==============================
Subjects: Dp 29–5

Development of an Innovation Procurement Framework for Clinicians

Angela Coderre-Ball Queen’s University

Nancy Dalgarno Queen’s University

Jessica Baumhour Queen’s University

Iris Ko Georgian College

Vittoria Zubani Queen’s University

Electronic publication date: 2020 Apr 18

JOURNAL INFORMATION
==============================
NLM Title Abbreviation: Can Med Educ J
Journal ID: Can Med Educ J
NLM Journal ID: 101560935
Journal ID: CMEJ

Canadian Medical Education Journal

EISSN: 1923-1202
Publisher: University of Saskatchewan

ARTICLE INFORMATION
==============================
Subjects: Dp 29–6

Démarche d'intégration des patients partenaires en évaluation des étudiants médecine et en sciences de la santé. L'exemple des étudiants en médecine en fin d'externat à l'Université de Montréal.

Clara Dallaire Université de Montréal

Philippe Karazivan Université de Montréal

Marie-Pierre Codsi Université de Montréal

Annie Descôteaux Université de Montréal

Mathieu Jackson Université de Montréal

Audrey L'Espérance Centre d'excellence sur le partenariat avec les patients et le public

Electronic publication date: 2020 Apr 18

JOURNAL INFORMATION
==============================
NLM Title Abbreviation: Can Med Educ J
Journal ID: Can Med Educ J
NLM Journal ID: 101560935
Journal ID: CMEJ

Canadian Medical Education Journal

EISSN: 1923-1202
Publisher: University of Saskatchewan

ARTICLE INFORMATION
==============================
Subjects: Dp 29–7

From Learner to Scholar: Diversifying the Pool of Medical Educators through a PGME Track

Taryn Taylor Emory University School of Medicine

Ulemu Luhanga Emory University School of Medicine

Electronic publication date: 2020 Apr 18

